# Dynamic establishment and maintenance of the human intestinal B cell population and repertoire following transplantation in a pediatric-dominated cohort

**DOI:** 10.3389/fimmu.2024.1375486

**Published:** 2024-06-28

**Authors:** Jianing Fu, Thomas Hsiao, Elizabeth Waffarn, Wenzhao Meng, Katherine D. Long, Kristjana Frangaj, Rebecca Jones, Alaka Gorur, Areen Shtewe, Muyang Li, Constanza Bay Muntnich, Kortney Rogers, Wenyu Jiao, Monica Velasco, Rei Matsumoto, Masaru Kubota, Steven Wells, Nichole Danzl, Shilpa Ravella, Alina Iuga, Elena-Rodica Vasilescu, Adam Griesemer, Joshua Weiner, Donna L. Farber, Eline T. Luning Prak, Mercedes Martinez, Tomoaki Kato, Uri Hershberg, Megan Sykes

**Affiliations:** ^1^ Columbia Center for Translational Immunology, Department of Medicine, Columbia University, New York, NY, United States; ^2^ Department of Human Biology, University of Haifa, Haifa, Israel; ^3^ Department of Pathology and Laboratory Medicine, University of Pennsylvania, Philadelphia, PA, United States; ^4^ Department of Pathology, Columbia University, New York, NY, United States; ^5^ Department of Pediatrics, Columbia University, New York, NY, United States; ^6^ Department of Microbiology and Immunology, Columbia University, New York, NY, United States; ^7^ Division of Digestive and Liver Diseases, Department of Medicine, Columbia University, New York, NY, United States; ^8^ Department of Surgery, Columbia University, New York, NY, United States

**Keywords:** intestinal transplantation, B cell repertoire sequencing, resident memory B cells, B cell subpopulations, human longitudinal studies

## Abstract

**Introduction:**

It is unknown how intestinal B cell populations and B cell receptor (BCR) repertoires are established and maintained over time in humans. Following intestinal transplantation (ITx), surveillance ileal mucosal biopsies provide a unique opportunity to map the dynamic establishment of recipient gut lymphocyte populations in immunosuppressed conditions.

**Methods:**

Using polychromatic flow cytometry that includes HLA allele group-specific antibodies distinguishing donor from recipient cells along with high throughput BCR sequencing, we tracked the establishment of recipient B cell populations and BCR repertoire in the allograft mucosa of ITx recipients.

**Results:**

We confirm the early presence of naïve donor B cells in the circulation (donor age range: 1-14 years, median: 3 years) and, for the first time, document the establishment of recipient B cell populations, including B resident memory cells, in the intestinal allograft mucosa (recipient age range at the time of transplant: 1-44 years, median: 3 years). Recipient B cell repopulation of the allograft was most rapid in infant (<1 year old)-derived allografts and, unlike T cell repopulation, did not correlate with rejection rates. While recipient memory B cell populations were increased in graft mucosa compared to circulation, naïve recipient B cells remained detectable in the graft mucosa for years. Comparisons of peripheral and intra-mucosal B cell repertoires in the absence of rejection (recipient age range at the time of transplant: 1-9 years, median: 2 years) revealed increased BCR mutation rates and clonal expansion in graft mucosa compared to circulating B cells, but these parameters did not increase markedly after the first year post-transplant. Furthermore, clonal mixing between the allograft mucosa and the circulation was significantly greater in ITx recipients, even years after transplantation, than in deceased adult donors. In available pan-scope biopsies from pediatric recipients, we observed higher percentages of naïve recipient B cells in colon allograft compared to small bowel allograft and increased BCR overlap between native colon vs colon allograft compared to that between native colon vs ileum allograft in most cases, suggesting differential clonal distribution in large intestine vs small intestine.

**Discussion:**

Collectively, our data demonstrate intestinal mucosal B cell repertoire establishment from a circulating pool, a process that continues for years without evidence of stabilization of the mucosal B cell repertoire in pediatric ITx patients.

## Introduction

The gastrointestinal (GI) tract is increasingly recognized as a site of abundant and diverse immune system representation and responsiveness. Additionally, immune responses local to the gut impact the entire body through systemic immune axes with the neuroendocrine system (1). The local microbiome milieu critically shapes gut lymphocytes and immune responses (2), with commensal and pathogenic microbes in the gut environment shaping the B cell repertoire (3, 4). The composition of gut and gut-trafficking immune cells, and their relationships with cells in other sites, continue to be catalogued and their impacts both locally and peripherally are increasingly described, including in both infectious and autoimmune disease (5, 6) as well as in transplantation (7–10). Recently, deep immune repertoire profiling of B cell clones from adult humans established that B cell clones partition into two major networks, one in the GI tract and one in the blood, bone marrow, spleen, and lung (11). Gut populations include many specialized B cell subsets, such as marginal zone B cells (12) and recently reported B resident memory (BRM) cells (13). A study on the maturation of human intestinal B cell responses during fetal development showed that diverse intestinal B cell receptor (BCR) profiles exist in the early 2^nd^ trimester and that BCR repertoires show increasing heavy chain CDR3 length with advancing gestational age (14). However, how gut B cell populations and BCR repertoires are established and maintained over time in humans remains largely unknown. Such information could improve understanding of alloimmune, autoimmune and pathogen-specific immune responses in the gut.

Intestinal transplantation (ITx) provides a unique opportunity to map the dynamic establishment of gut B cell populations and BCR repertoires through the serial ileal allograft biopsies taken for clinical surveillance of rejection. Although intestinal transplants are matched for blood type in most cases, they are not matched for human leucocyte antigens (HLA), allowing discrimination of donor and recipient cells in both the periphery and within the allograft by HLA alleles (15). ITx is used to treat patients with life-threatening complications of irreversible intestinal failure (16). Despite lymphodepleting induction therapy, high rejection rates limit transplant success and graft survival remains about 50% after 5 years (17). Infection (18), post-transplant lymphoproliferative disease (19) and graft-versus-host disease (GVHD) (20, 21) also cause significant morbidity and mortality. Our previous studies suggest that the higher proportion of donor to recipient lymphocytes leads to stronger graft-versus-host (GvH) responses compared with host-vs-graft (HvG) responses, and thereby associates with less rejection and better clinical outcomes in ITx (22–25). Peripheral blood chimerism that includes naïve B cells is associated with engraftment of donor graft-derived hematopoietic cells in the recipient’s bone marrow and the presence of donor graft-derived GvH-reactive effector T cells that may make “space” in the marrow for the donor hematopoietic cells (23–25). The higher donor lymphoid cell load in multivisceral transplant (MVTx) recipients is expected to result in stronger GvH responses than in isolated ITx (iITx) recipients. This difference may also impact B cell responses, as reduced donor-specific antibody (DSA) development is observed after liver-inclusive transplants compared with iITx (26, 27) and *de novo* DSA development is associated with increased graft loss due to rejection (28).

Given the importance of intestinal lymphocytes in controlling infection and immune responses, intra-graft lymphocyte composition, reactivity, and function are likely important determinants of ITx outcomes. In depth analysis of intestinal T cells, hematopoietic stem and hematopoietic progenitors, and innate lymphoid cells, including their turnover and responses in association with clinical outcomes following ITx, have provided major insights into these relationships (22–25, 29, 30). Our previous studies showed that within the allograft, while donor CD45^-^ epithelial cells were retained (23), some innate lymphoid cells persisted long term (24, 29), CD56^+^CD3^-^ NK cells turned over relatively quickly (23), and CD3^+^ lymphocytes persisted for variable amounts of time in relation to rejection (23) and donor age, with more rapid replacement by recipient T cells in association with rejection or younger donor age (<1 year old) (25).

B cells may participate in rejection of solid organ transplants both by serving as antigen-presenting cells for T cells and by producing alloantibodies in response to T cell help (31, 32). B cells can also function as regulatory populations, with recent identification of regulatory B cell (Breg) markers TIM-1 and IL-10 in both mice and humans (33–35). Nevertheless, intra-graft B cell dynamics and the maturation of B cells at both the phenotypic and clonal levels have not been analyzed in detail after human solid organ transplantation. We therefore performed in-depth B cell phenotyping and sequencing analysis on serial biopsies and peripheral blood samples collected after transplant, primarily in the absence of rejection. Our B cell phenotyping and repertoire analyses enhance an understanding of gut mucosal B cell dynamics in relation to donor age and circulating B cell populations after transplantation. These studies provide a cornerstone for future investigations on donor-reactive and tolerogenic B cell immune cell responses and pathologic consequences for the graft.

## Methods

### Study approval

The intestinal transplant protocol was approved by the Columbia University Institutional Review Board (IRB nos. AAAJ5056, AAAF2395, and AAAS7927). All subjects or legal guardians provided their written, informed consent and assent (**Supplementary Table S1**). Healthy control blood and ileum tissues were obtained from deceased organ donors through a collaboration and research protocol with LiveOnNY, the organ procurement organization for the New York metropolitan area as previously described (36) (**Supplementary Table S2**). The use of deceased organ donor tissues is not human subjects research, as confirmed by the Columbia University IRB.

### Human intestinal transplant patient recruitment and clinical protocols

Our study involves 27 intestinal transplants, including 3 re-transplants (Pt4, Pt16, Pt21) indicated as Pt4 reTx, Pt16 reTx, and Pt21 reTx. Detailed B cell phenotyping studies of 13 transplants were performed and are reported here, including Pt14, Pt17, Pt19 through Pt27, Pt4 reTx, Pt16 reTx, and Pt21reTx (**Supplementary Table S1**). All recipients studied except Pt22 and Pt26 were pediatric (under 15 years old with a median age of 3 years at the time of transplant. Protocol and for-cause endoscopic allograft biopsies were obtained as described previously (22–25) in conjunction with clinical surveillance in the post-ITx period. Graft rejection was graded as negative, indeterminate, mild, moderate or severe based on the reported histopathologic scoring scheme (37). Blood samples were collected up to 4 times during the first month after Tx and thereafter at least once per month when available. All patients received anti-thymocyte globulin (ATG) induction therapy (total dose: 6–10 mg/kg) followed by a maintenance regimen of long-term tacrolimus and steroids. Tacrolimus was initiated on day 1, adjusted for a target trough level of 15 to 20 ng/mL during the first month after Tx, and gradually tapered down to a maintenance level of 10 to 15 ng/mL for 2–6 months after Tx, and further tapered down to 7–10 ng/mL thereafter. Patients received tapering doses of methylprednisolone from day 0 to day 5 after Tx, after which prednisone was maintained until indicated by the transplant team and tapered off by 6 to 12 months. Allograft rejections were treated with augmented immunosuppression based on the severity of rejection.

### Lymphocyte isolation from intestinal mucosa

We initially observed that B cells are predominately distributed in lamina propria rather than intraepithelially in the human intestinal mucosa (data not shown), consistent with observations in mice (38). Therefore, we applied one-step Collagenase D incubation for combined intraepithelial lymphocytes (IELs) and lamina propria lymphocytes (LPLs) isolation from graft biopsy specimens, according to a protocol adapted from our previous reports (15). Briefly, biopsy specimens were digested by stirring in flasks containing 25–50 mL collagenase-containing medium (RPMI 1640, 1 mg/mL Collagenase D, 100 IU/mL penicillin-streptomycin) for 1 hour in a 37°C water bath. After passing through a 40 µm filter, cells were washed with MLR media (RPMI 1640, 5% human serum, Hepes 10 mM, 2-ME 50 µM) before proceeding to subsequent assays. Larger full thickness surgical specimens obtained at the time of stoma closure/revision were prepared first by washing of the mucosal layer in PBS, followed by separation of the mucosal layer and isolation of IELs for other uses, prior to incubation for LPLs. For IEL isolation, minced pieces of mucosa were incubated for 30 min–1 hr in flasks first containing 2 mM dithiothreitol and 0.5 mM EDTA, followed by 0.5 mM EDTA, both under continuous stirring over a 37°C water bath. LPLs were isolated from the remaining tissue, digested and stirred in collagenase-containing medium as described above for 1.5–2 hours in a 37°C water bath. DNAse (0.1 mg/mL) and Fungizone (1 µg/mL) were added to media when large specimens were processed. PBMCs were isolated from approximately 0.5–10 mL samples of whole blood by fractionation over Histopaque gradients, followed by ACK lysis of remaining red blood cells when necessary.

### HLA-specific cell surface staining

Candidate HLA class I allele group-specific monoclonal antibodies (mAbs) were screened for the ability to discriminate donor and pre-transplant (pre-Tx) recipient cells, based on clinically available molecular HLA typing information. Each HLA-specific antibody was used in combination with pan–HLA-ABC antibody and quality control (QC) tested for specificity (15). Those that readily distinguished donor from the pre-Tx recipient PBMCs were included in lineage-specific panels of antibodies (**Supplementary Table S3**), as reported previously (22–25). There were no closely-related HLA genotypes between donors and recipients in the cohort analyzed, and we were always able to find at least one HLA-A or B allele group that successfully distinguished donor cells from recipient cells. If the staining intensity was dim when using one HLA-allele group specific mAb for either the donor or recipient, we used a second HLA-allele group specific mAb validated by HLA-QC to stain both donor and recipient cell populations to more clearly distinguish donor and recipient cells in two-color plots. HLA-specific antibodies used in this study are summarized as follows: HLA-ABC APC (G46–2.6, BD Biosciences, catalog 555555), HLA-ABC PE (G46–2.6, BD Biosciences, catalog 555553), HLA-ABC BV786 (G46–2.6, BD Biosciences, catalog 740982), HLA-A02/28 PE (BB7.2, BD Biosciences, catalog 558570), HLA-A02/28 FITC (One Lambda, catalog FH0037), HLA-A02/28 Biotin (One Lambda, catalog BIH0037), HLA-A09 FITC (One Lambda, catalog FH0964), HLA-A09 Biotin (One Lambda, catalog BIH0964), HLA-A03 APC (eBioscience, catalog 17–5754-42), HLA-B08 FITC (One Lambda, catalog FH0536A), HLA-B12 FITC (One Lambda, catalog FH0066), HLA-A30/31 Biotin (One Lambda, catalog BIH0067), HLA-B27 FITC (One Lambda, catalog B27F50X).

### Flow cytometry

To check B cell subset frequencies in tissue samples, cells were resuspended at 1x10^6^ cells per 100 μL in FACS Buffer containing 0.1% BSA and 0.1% Sodium Azide in HBSS. Cellular staining was performed after human Fc block (Fc1.3216, BD Biosciences, catalog 564220), using combinations of the following antibodies plus HLA-specific mAbs as above, prior to DAPI staining to identify dead cells: CD3 APC-Cy7 (SK7, BD Biosciences, catalog 561800), CD14 APC-Cy7 (HCD14, BioLegend, catalog 325620), CD33 APC-Cy7 (P67.6, BioLegend, catalog 366614), CD326 APC-Cy7 (9C4, BioLegend, catalog 324245), CD19 BUV496 (SJ25C1, BD Biosciences, catalog 612938), CD20 FITC (L27, BD Biosciences, catalog 347673), CD20 APC (2H7, BD Biosciences, catalog 559776), CD38 PE-Cy7 (HIT2, BioLegend, catalog 303516), CD27-BV711 (0323, BioLegend, catalog 302833), IgM PE-CF594 (G20–127, BD Biosciences, catalog 562539), IgG V450 (G18–145, BD Biosciences, catalog 561299), IgD BV605 (IA6–2, BioLegend, catalog 348232), IgA APC (IS11–8E10, Miltenyi Biotec, catalog 130–093-073), IgA Biotin (G20–359, BD Biosciences, catalog 555884), CD21 PerCP-Cy5.5 (Bu32, BioLegend, catalog 354908), CD24 BUV395 (ML5, BD Biosciences, catalog 563818), CD45RB PE (MEM-55, BioLegend, catalog 310204), CD69 BV650 (FN50, BioLegend, catalog 310934), CD138 AF700 (MI15, BioLegend, catalog 356512), SA BUV737 (BD Biosciences, catalog 612775). Data were acquired using an LSR II flow cytometer (BD Biosciences) with DIVA software. Analysis was carried out using FlowJo (BD Biosciences), Prism (GraphPad by Dotmatics), and Tableau (Salesforce) software. There was no selection bias for patients and samples undergoing in-depth B cell phenotyping analysis. Samples used for B cell phenotyping represented approximately 1/3 of total follow up biopsies we obtained for research, as the remaining 2/3 of biopsies were used for T cell phenotyping and repertoire studies that have been published separately (23–25, 30, 39).

### Cell sorting

To isolate recipient B cells from tissues, cell surface staining was performed using combinations of the following mAbs plus HLA-specific mAbs as above, after human Fc block (Fc1.3216, BD Biosciences, catalog 564220) and prior to DAPI staining to identify dead cells: CD45 V500 (HI30, BD Biosciences, catalog 560777), CD3 Per- CP-Cy5 (UCHT1, BD Biosciences, catalog 552852), CD19 BV650 (HIB19, BioLegend, catalog 302238), CD138 FITC (DL-101, BioLegend, catalog 352304), CD138 APC (DL-101, BioLegend, catalog 352308), Streptavidin PE-Cy7 (BD Biosciences, catalog 557598). Cell sorting was accomplished using an Influx cell sorter (BD Biosciences) and DIVA software. Sorted DAPI^-^ CD45^+^ CD3^-^ recipient HLA^+^ CD19^+^ and/or CD138^+^ cells were preserved in Puregene cell lysis solution (Qiagen, catalog 158906) and maintained at room temperature prior to subsequent DNA purification.

### B cell cultures and DSA assay

For *in vitro* stimulation of B cells and plasma cells, CD19^+^ and/or CD138^+^ cells as above were sorted directly into medium containing 10% FBS (Gemini, catalog 100–106), 2 mM L-Glutamine (Gibco, catalog 25030149), 50 uM 2-ME (Sigma-Aldrich, catalog M3148), 100units/mL Penicillin and 100 μg/mL Streptomycin (Gibco, catalog 15140148) in IMDM (Gibco, catalog 12440061). After washing, cells were resuspended for culture at 0.1x10^6^ cells/mL with 600 IU/mL IL-2 (R&D Systems, catalog 202IL010), 500 ng/mL anti-CD40 (BioLegend, catalog 334304), 25 ng/mL IL-10 (PeproTech, catalog 200–10), 2.5 μg/ml CpG (Hycult, catalog HC4039), 100 ng/mL IL-21 (PeproTech, catalog 200–21) (40, 41) for 7 days in an incubator controller setup maintaining 3% O_2_ (BioSpherix, catalog P360). Supernatants were concentrated approximately 20-fold using Amicon Ultra-4 Centrifugal Filters (Millipore, Catalog UFC805024). The presence of DSA in concentrated supernatants was determined by the clinical laboratory using the LabScreen Single Antigen Bead Luminex assay (One Lambda) according to the vendor’s protocol and analysis HLA Visual Software. The mean fluorescence intensity (MFI) values of beads containing donor mismatched antigens for each locus, HLA-A, HLA-B, HLA-C, HLA-DR, HLA-DP, and HLA-DQ were analyzed. For research purposes, samples in which single antigen bead IgG staining exceeded the normalized MFI baseline by 2,000 MFI (for serum) or a culture medium control by 500 MFI (for concentrated supernatants) were considered to show specificity.

### Sequencing and library preparation

Genomic DNA was extracted from FACS-sorted recipient HLA^+^ CD19^+^ and/or CD138^+^ cells from post-Tx biopsies and blood samples using the Qiagen Gentra Puregene blood kit (Qiagen, Valencia, CA, Cat. No. 158389) as previously described (11). DNA quality and yield were evaluated by spectroscopy (Nanodrop, ThermoFisher Scientific, Waltham, MA). PCR amplification of immunoglobulin heavy-chain gene rearrangements from genomic DNA samples using primers in framework region 1 and joining region was performed as previously published (42, 43). DNA extraction and BCR sequencing were carried out in the Human Immunology Core Facility at the University of Pennsylvania. Experiments were performed using the Illumina 2 x 300-bp paired-end kits (Illumina MiSeq Reagent Kit v3, 600-cycle, Illumina MS-102–3003).

### Quality control and germline and clonal annotation of raw sequence data

pRESTO (44) was used for quality control, with a protocol similar to that previously described (11, 45). Germline annotation and V and J assignment was done using IgBLAST (43). Sequences were trimmed to IMGT position 80 (the beginning of CDR1), in order to exclude primers and imported to immuneDB. ImmuneDB (46, 47) was then used to combine sequences sharing the same VH and JH genes that had the same CDR3 length and at least 85% amino acid similarity in the CDR3. Adult sequencing data, which were used for comparisons, was taken from a previous study that used similar protocols for sequencing and quality control (11). All data were re-annotated exactly as described above, to be comparable.

### Rejection samples

**Figure 1:**
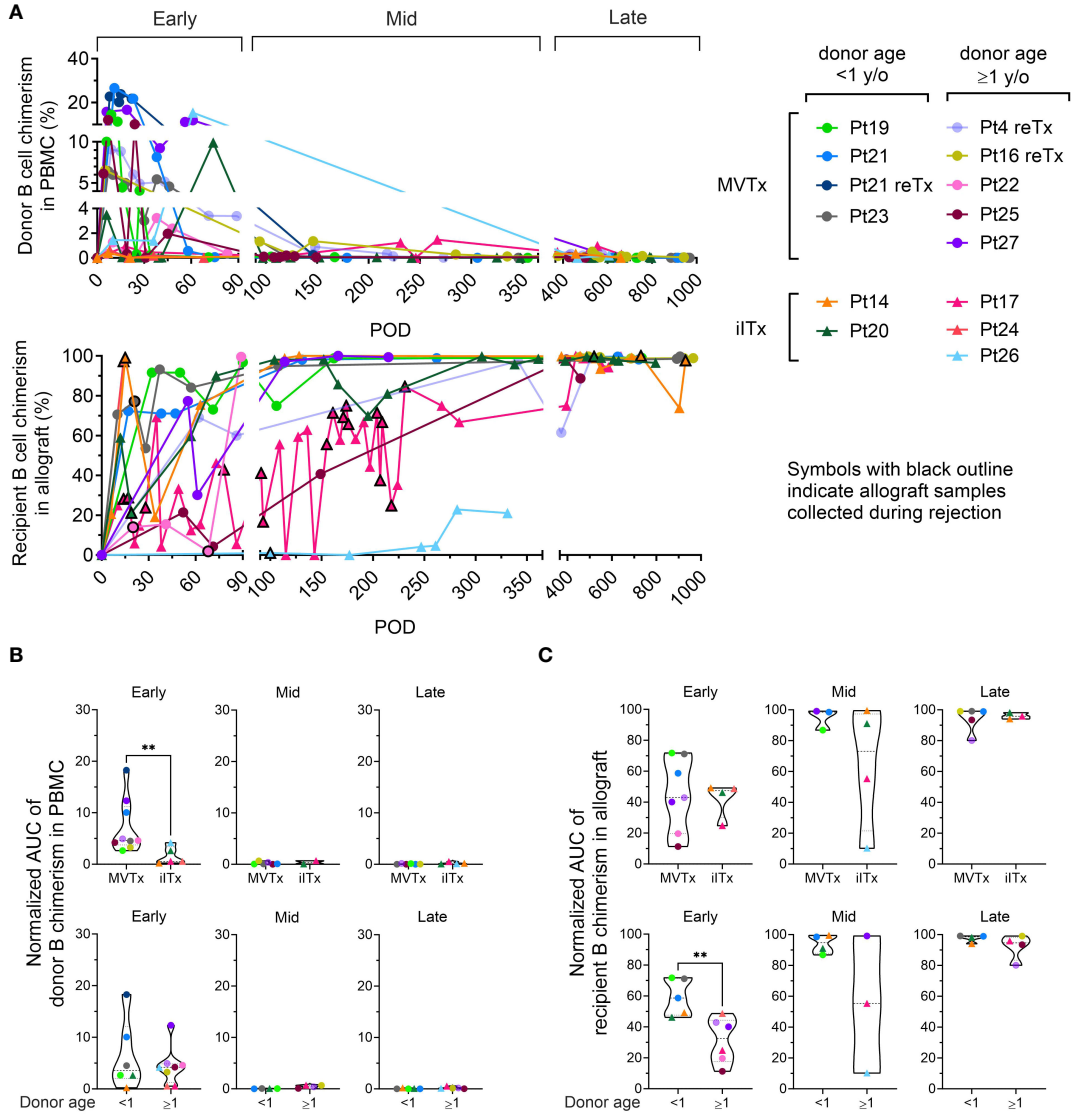
B cell chimerism in PBMCs and intestinal allografts post ITx and correlation with transplant type and donor age. **(A)** Chimerism of donor CD19^+^ B cells in recipient PBMC and recipient CD19^+^ B cells in ileum allograft post-Tx. Samples containing at least 40 cells in the parent gate (CD19^+^ B cells) are shown. MVTx patients are represented by circles and iITx represented by triangles. Symbols with black outline in recipient B cell chimerism in allograft plots indicate samples collected whose histology demonstrated mild to severe ACR, or concurrance of DSA^+^ AMR. Area under the curve (AUC) values normalized by days of measurement (POD_last_ – POD_first_) of donor B cell chimerism in PBMC plots **(B)** and recipient B cell chimerism in ileal allograft plots **(C)** during early (POD0–90), mid (POD91–365) and late (POD366–1000) post-Tx periods were subgrouped by MVTx vs iITx (upper panels) and donor age <1 vs ≥1 year old (lower panels). For **(B, C)** dashed lines from the bottom to the top within each condition represent the first quartile, median, and third quartile of data distribution, respectively. The Mann-Whitney U test was performed to determine statistical significance (**p<0.01).

Samples that were determined to display any level of rejection, including mild or greater acute cellular rejection (ACR), or concurrence of DSA^+^ antibody-mediated rejection (AMR) via histology and serum DSA measures (MFI ≥ 2,000), were either identified specifically as in **Figure 1A** (associated with **Supplementary Figure 2**) or excluded from the analyses for reasons given in the Results section.

### Clone filtering

For different analyses, clones were filtered by tissue type and by size. A clone was defined as detectable in a tissue if it contained at least one functional sequence (with a copy number of at least 2) in that tissue. To determine clone size within a time bracket, the number of unique instances of a clone was summed for all samples within the time bracket (42, 48). Clones were also sometimes filtered by the number of nodes observed in their lineages that were populated by a unique sequence. Finally, we defined trunk clones as clones having 5 or more unique mutations shared by at least 85% of their unique sequences.

### Measures reported

#### Mutation frequency

The mutation frequency of a sequence was calculated by dividing the total number of mutations found in the v gene by the sequenced length of the v gene. In our study, this metric is calculated with ImmuneDB (46, 47) and has the field name ‘v_mutation_fraction’. The average mutation frequency of a clone was calculated by averaging the v_mutation_fraction values across all unique instances in a clone.

#### Clumpiness

Clumpiness (49) quantifies how mixed two metadata labels (in this study blood and ileum or ileum and colon are the metadata labels used for clumpiness) are within a hierarchical lineage, with a value of 0 representing labels separated from each other on different branches, and a value of 1 representing labels highly mixed together within branches. We used this metric to determine the extent of mixing between unique sequences from different tissues within lineages. Clumpiness was calculated with https://github.com/DrexelSystemsImmunologyLab/clumpiness.

#### Diversity

Diversity of clones was calculated with https://github.com/DrexelSystemsImmunologyLab/diversity as previously described (50). Diversity is a quantification related to the number of unique clones in a sample and can be calculated with different “orders”. The higher the order, the more weight is given to large clones and less weight given to small clones when calculating diversity. At order 0, diversity is equal to the richness of the sample; in other words, the diversity is equal to the total number of unique clones in the sample, regardless of clone size. At higher orders of diversity, smaller clones contribute less to the diversity of the sample. A diversity order of 1 is commonly used when calculating diversity. Evenness is a term used in ecology when describing diversity relative to the total number of species in a population (51). Evenness has been previously described as a measure of B cell diversity (52). In this study, evenness of a repertoire was defined as the diversity of clones at order 1 normalized by their richness (the number of unique clones in a population). This measure in effect expresses diversity of clone sizes (or clonality) without being affected by the different number of unique clones between patients/data points.

#### Cosine similarity

Cosine similarity was calculated with the python library sklearn.metrics.pairwise.cosine_similarity (scikit-learn 1.4.2). Cosine similarity is a commonly used metric to quantify how similar two documents are, or in our case, how similar two repertoires of clones are to each other. It is advantageous because it ignores differences in scale, which is a common issue when comparing two BCR repertoires. We used clone size (the sum of unique instances found in a clone among all samples within a given tissue and POD bracket) as the parameter for cosine similarity, as described previously (11). If the relative sizes of the clones that span both repertoires remain similar between each repertoire, the cosine similarity value will be close to one. Conversely, if the relative sizes of clones from one repertoire vary widely from the other repertoire, the cosine similarity value will be close to zero.

### Statistics

We used the nonparametric Mann-Whitney U test (two-sided) whenever we compared two independent samples (e.g., when comparing data points between children and adults). A log-rank (Mantel-Cox) test was performed for the Kaplan-Meier plot of freedom from moderate or severe rejection of patients with or without *de novo* Class I/II DSA in serum. To identify if there was a consistent direction in changes of the size of individual clones across two time points, we performed a sign test between each two time points, where each clone present at both time points that contained more unique instances at the later time bracket was given a + and each clone that contained fewer instances in the later time bracket was given a -. Clones that did not change in size were excluded from the analysis. In all cases, p-values < 0.05 were considered significant.

### Data and materials availability

Raw BCR-seq data in FASTA format is available at Sequence Read Archive (SRA: https://www.ncbi.nlm.nih.gov/sra) under accession number PRJNA1031101. The code written in Python (version 3.5+) used to analyze BCR-seq data is available in the GitHub repository at https://github.com/DrexelSystemsImmunologyLab/Pediatric_gut_homeostatsis_paper. Requests to transfer human biospecimens outside of the organization must be submitted for Columbia’s IRB review and approval.

## Results

### B cell chimerism in tissues post ITx and correlation with type of transplant, donor age, rejection and DSA

To determine the dynamics of recipient and donor B cell populations in serial blood and allograft mucosal biopsy samples, we used surface HLA-specific staining to distinguish recipient and donor cells for each transplant (**Supplementary Figure 1**, **Supplementary Table S3**) in three post-Tx periods (**Figure 1A**): early (post operative day [POD]0–90), mid (POD91–365) and late (POD366–1000). Donor B cell chimerism was predominately detected in the blood in the early post-Tx phase, with higher peak levels and longer persistence in MVTx compared to iITx recipients, as reflected by the area under the curve (AUC) values normalized by days of measurement (POD_last_ – POD_first_) (**Figure 1B**). B cell chimerism declined in the mid and late post-Tx phases. These observations concur with our previous report that multilineage chimerism in blood, including B cell chimerism, is more dominant in MVTx than iITx patients (24).

Recipient B cell replacement of donor B cells in the intestinal graft was variable and fluctuated over time. The majority of patients demonstrated consistent replacement of mucosal B cells by the recipient of at least 80% within 1 year post-Tx (**Figure 1A**). In the allograft, the normalized AUC of recipient B cell chimerism in the early-stage post-Tx time period was significantly greater in patients with younger (<1 year old) than older (≥1 year old) donors, regardless of the type of Tx (**Figure 1C**).

In contrast to observations regarding recipient T cell replacement of donor T cells in mucosal allografts (23, 25), we detected no significant correlation between the rate of recipient replacement of donor B cells in the graft and the presence of significant rejection, including mild or greater ACR and/or AMR, in the early period post-Tx (**Supplementary Figure 2A**). The remaining analysis in our study was focused on non-rejecting samples because: 1) Our allograft sample collection was highly skewed towards non-rejecting time points in almost all patients except Pt17 (**Figure 1A** lower panel: symbols with black outline indicate allograft samples collected during rejection); 2) For some patients, B cell phenotyping data were only available at a few late post-Tx time points, such as Pt14 POD695 PBMC and Pt17 POD640 ileal biopsy.

We detected no significant correlation between the rate of recipient replacement of donor B cells in the graft and the development of *de novo* Class I and/or II DSA in serum in the early post-Tx period (**Supplementary Figure 2B**). However, our data from this small cohort of patients recapitulate previous observations (26) that *de novo* development of class I and class II DSA correlates with higher rates of moderate or severe ACR (**Supplementary Figure 3A**). To assess the possibility that DSA might be produced within the allograft, a single mucosal sample containing sufficient B cells to permit *in vitro* culture allowed us to demonstrate local production of DSA by mucosal recipient B cells. This DSA matched the specificity detected in the serum on the same day in this patient (Pt14 POD1764), when the graft was explanted due to chronic rejection following persistent acute rejection (**Supplementary Figure 3B**). This result suggests that recipient B cells within the allograft may give rise to DSA following ITx.

### B cell subset chimerism in the peripheral blood and allograft over time and correlation with donor age

**Figure 2:**
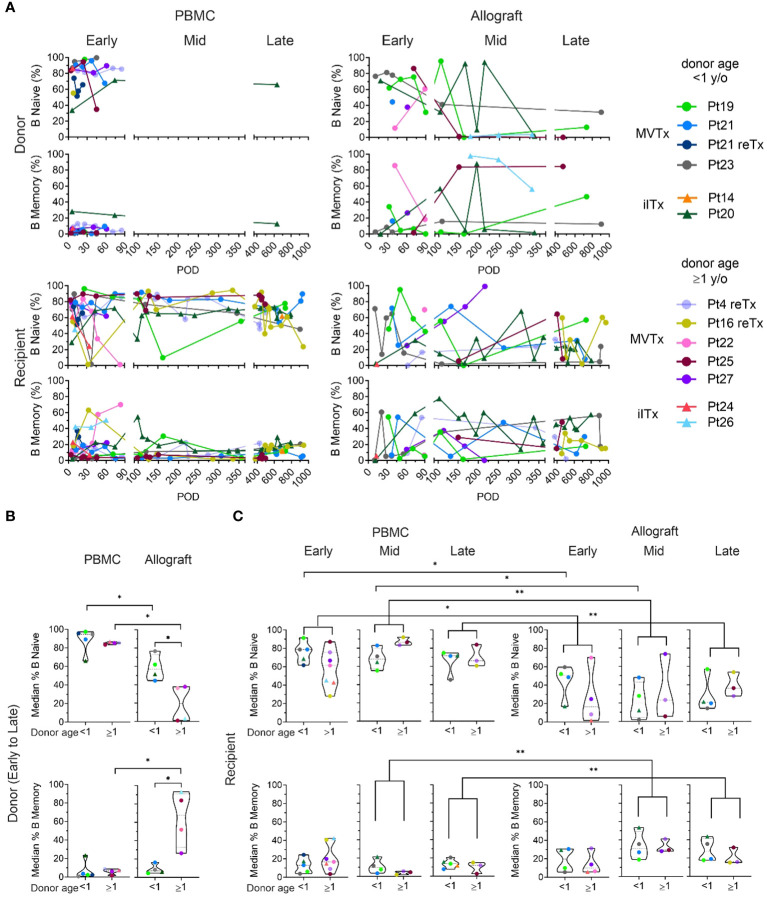
Naive and memory B cell chimerism in the PBMC and intestinal allograft over time. **(A)** Percentages of donor and recipient naive (CD27^-^IgD^+^) and memory B cells (CD27^+^ IgD^+/-^) in PBMC and ileal allograft during quiescence in post-Tx time brackets defined as in **Figure 1**. The median percentage among samples for each patient in a given period (represented by the median of the timepoints sampled) are shown for **(B, C)**. **(B)** Median donor naïve and memory B cell percentages among total donor B cells in PBMC and ileal allograft from early to late post-Tx periods were subgrouped by donor age. **(C)** Median recipient naïve and memory B cell percentages of total donor B cells in PBMC and ileal allograft during early, mid and late post-Tx periods were subgrouped by donor age. For **(B, C)** dashed lines from the bottom to the top within each condition represent the first quartile, median, and third quartile of data distribution, respectively. The Mann-Whitney U test was performed to determine statistical significance (*p<0.05, **p<0.01).

To further characterize B cell populations after ITx, we used multicolor flow cytometry to identify naïve (CD27^-^IgD^+^) and memory (CD27^+^IgD^+/-^) subsets among recipient and donor B cells (**Supplementary Figures 1**, **2**). From the early to the late post-Tx period, donor B cells in the PBMC were mainly naïve. Percentages of naïve cells among donor B cells were generally higher in peripheral blood than in the allograft (**Figures 2A, B**). Within the allograft, younger donors (<1 year old) showed significantly higher proportions of naïve B cells and significantly lower levels of memory B cells compared to older donors (≥1 year old) (**Figure 2B**), demonstrating B cell maturation in the human gut after infancy.

Recipient B cells in the circulation and the allograft included both naïve and memory subsets, with no significant differences between younger vs older donor groups at early, middle or late post-Tx periods (**Figure 2C**). However, when patients with younger and older donors were combined, we found that recipient B cells within the allograft showed significantly lower median percentages of naïve cells and higher median percentages of memory cells compared to circulating recipient B cells, especially during the middle and late post-Tx periods, suggesting conversion of the initially-repopulating naïve recipient B cells or gradual entry into the mucosa of recipient memory B cells over time (**Figure 2C**). However, even in the late stage post-Tx, median percentages of graft mucosal recipient B cells with the memory phenotype remained less than 50%, in contrast to donor B cells when donors were at least one year old (**Figures 2B, C**).

**Figure 3:**
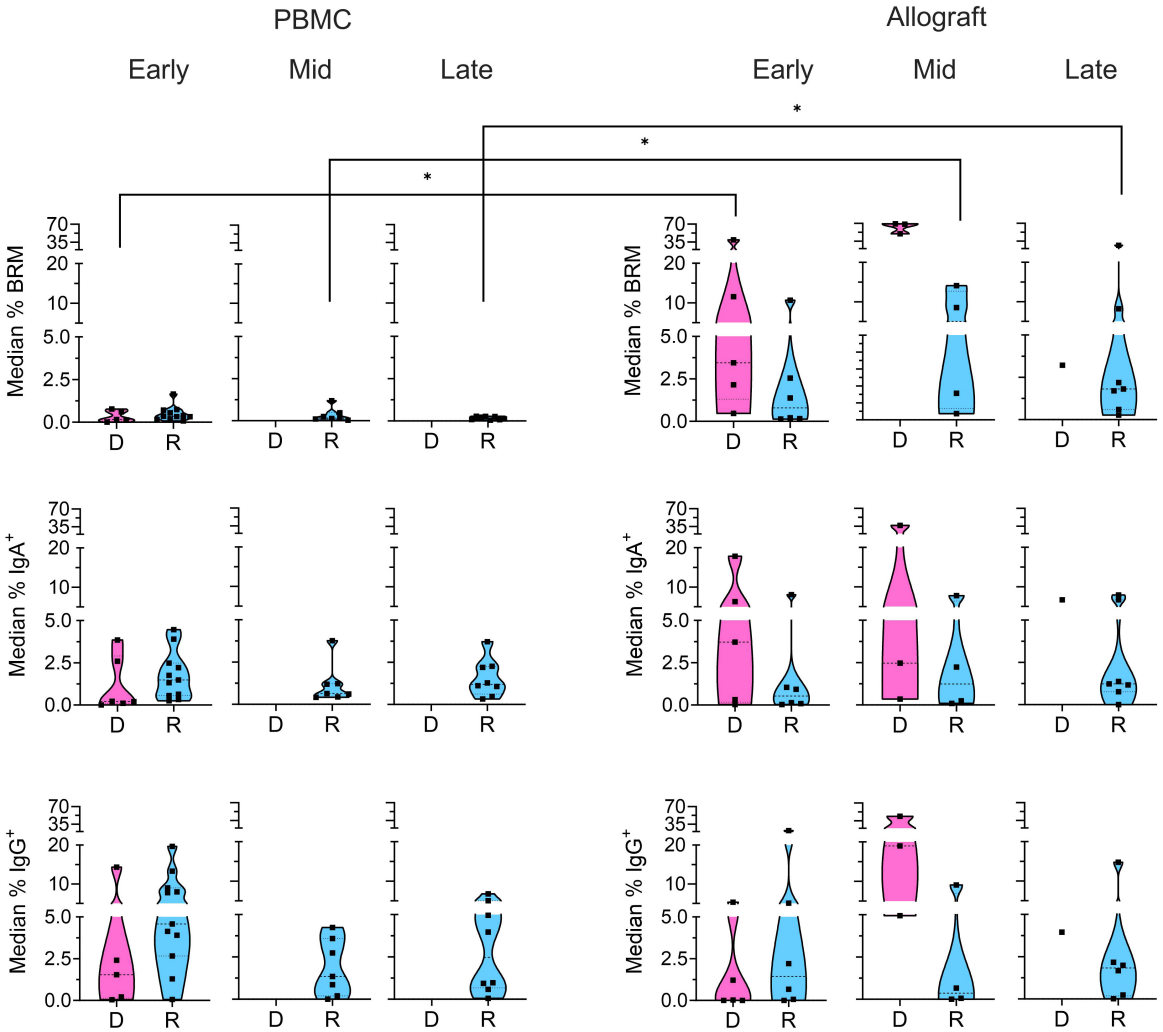
Memory subsets in donor and recipient B cells. Median percentages of donor (“D”: pink) and recipient (“R”: blue) BRM cells (CD69^+^CD45RB^+^), IgA^+^, and IgG^+^ B cells from the CD24^+^ memory gate (**Supplementary Figure 1**) within total recipient B cells for each patient in the PBMC and ileal allograft during early, mid and late post-Tx periods. In all cases, only quiescent samples exceeding a 40 cell threshold in the preceding parent gate are shown. Low sample numbers in the late time bracket for donor cells reflect near-complete replacement by recipient B cells at this stage. Dashed lines from the bottom to the top within each condition represent the first quartile, median, and third quartile of data distribution, respectively. The Mann-Whitney U test was performed to determine statistical significance (*p<0.05).

Resident memory B cells (BRM: CD69^+^CD45RB^+^) have recently been identified in human intestinal tissues (13). Our analysis revealed the presence of donor BRM in the intestinal allograft in all post-Tx periods (**Figure 3**). Recipient BRM also appeared in the allograft after transplant from early timepoints in some patients and were maintained through mid to late post-Tx periods at significantly higher levels than in circulating recipient B cells (**Figure 3**). Recipient memory B cells in the allograft also included class-switched IgA^+^ and IgG^+^ cells at comparable levels to circulating B cells throughout the early to late post-Tx periods (**Figure 3**), supporting potential local secretion of DSA. Given that the recipient CD138^+^ plasma cells within the antibody secreting cell (ASC) gate in the ileal allograft samples were limited by insufficient cell numbers (**Supplementary Figure 1B**), we were unable to pursue this analysis.

### Mutation levels of B cell clones in the blood and gut pre- and post-Tx

**Figure 4:**
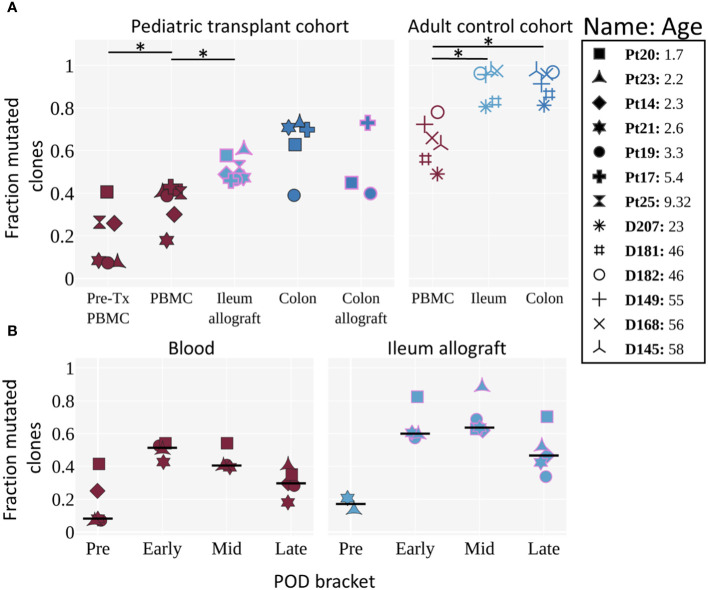
Mutation levels of BCR clones in pre- and post-Tx samples of pediatric ITx patients and adult deceased donor controls. **(A)** Fraction of mutated clones (clones with an average v gene mutation frequency >2%) by tissue for pediatric Tx patients and adult controls (11). Each individual is represented by a unique marker. The color of the marker represents the tissue, and the pink marker outline indicates that the tissue is an allograft. Tissues from the pediatric cohort are post-Tx unless specifically labeled as pre-Tx. Ages are shown in years. The Wilcoxon two-sided paired test was used to test for significance when the categories being compared were from the same cohort. An asterisk (*) denotes p-values <0.05. **(B)** Fraction of clones with an average v gene mutation frequency >2% by POD bracket for blood and ileum allograft (or native ileum for the “Pre” time bracket). Each POD is grouped into either pre, early, mid, or late time brackets (POD0, POD1–90, POD91–365 and POD>365, respectively). For each individual, the median among samples at a given POD bracket is shown. The horizontal black line represents the median among the individuals. POD samples were filtered for having >5 clones. Tissues are colored as in **Figure 4A**. **(A, B)** Pre-Tx samples represent native recipient blood/tissue and were taken during transplantation. Comparisons performed here were based on previous knowledge obtained in the adult control cohort and to determine: 1) whether tissue differs from blood in B cell mutation levels; and 2) whether mutation levels of circulating and ileal allograft B cells increase post-transplant. Our significance level is α = 0.05.

In order to investigate recipient B cell repertoire establishment over time in the intestinal allografts, high-throughput BCR heavy chain sequencing was performed on sorted recipient HLA^+^ B cells from serial biopsy specimens collected from pediatric ITx patients (**Supplementary Tables S4**, **S5**). Adult patients (Pts 22 and 26) were excluded from BCR-seq analyses due to limited post-Tx sample availability. We compared the samples from our pediatric ITx recipients to those from a cohort of deceased adult organ donors (11). Unfortunately, the limited size of the biopsies obtained per timepoint did not provide sufficient numbers of BCR clones within individual post-Tx specimens to allow analysis of clonal overlap over time or between tissues for the pediatric cohort. However, we were able to evaluate the overall progression of clonal mutation (see Methods) by combining data from multiple time points in the early (0–90 days), middle (91–365 days) and late (>365 days) time brackets (**Figure 4**). Mutation rates were significantly lower in the ileal allograft specimens from pediatric recipients than in the ileum from normal adults (**Figure 4A**). In both cohorts, the mutation rates in circulating blood B cell clones were significantly lower than those in the ileum. Clones in children from pre-Tx blood samples were significantly less mutated than those detected in post-Tx blood samples (**Figure 4A**). For both children and adults, mutation levels in colon samples mirrored their ileum counterparts (**Figure 4A**).

Next, we looked at how clonal mutation levels changed over time (**Figures 4B**; **Supplementary Figure 4**). Differences between the ileum allograft and blood were apparent during all time brackets, although statistical significance was not reached, likely due to limited sample size. B cell clones in two available pre-Tx allograft samples (donor age <1 year old for both samples) were largely unmutated, while clones from post-Tx samples were generally more mutated, peaking in the early and middle time brackets, before demonstrating a declining trend in the late time bracket in both the allograft and the blood.

### Dynamic mapping of recipient B cell repertoire diversity in circulation and ileal allograft

**Figure 5:**
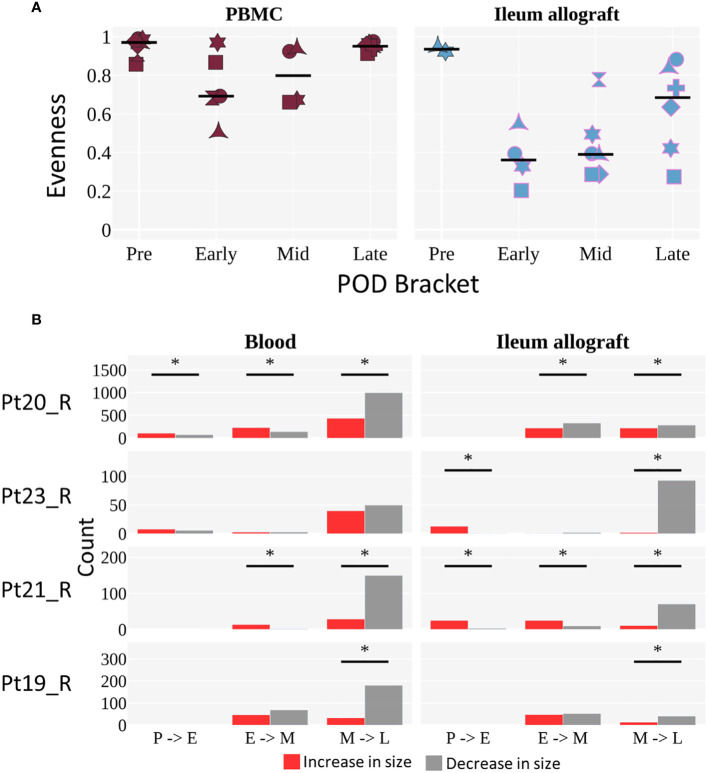
Diversity of B cell clones in peripheral blood and ileal allograft over time. **(A)** Evenness of clone distribution across time brackets. Evenness is calculated by dividing diversity (order 1) by richness (see Methods). The black horizontal line represents the median. Individuals are marked as in **Figure 4**. Tissues are colored as in **Figure 4A** (red for blood, light blue for ileum). The limited but novel and unprecedented data presented here exhibit a trend but are not sufficient to permit statistical analysis, as only two individuals span all 4 time points for ileum and those two individuals show differing trends. We therefore present the observed overall trends without attempting to generalize beyond the small population of individuals studied. **(B)** The number of clones that increase (red) or decrease (gray) in size (by number of unique instances) across two time brackets. The sign test was performed to determine significant difference in the direction of clonal growth between time brackets (*p <0.05). **(A, B)** Each POD is grouped into either pre (P), early (E), mid (M), or late (L) time brackets, as in **Figure 4B**.

To evaluate the progression of recipient B cell repertoire diversity over time, we assessed the evenness of clone distribution (see Methods), which is a measure of clonal diversity, by time bracket (**Figure 5A**). In some patients, the B cells in the blood showed a decline in evenness in the early or middle time brackets, then returned to the high pre-Tx levels in the late time bracket. In the ileum allograft, evenness was high pre-Tx, then declined in the early and middle time brackets, before increasing in the late bracket in 4 of the 6 patients (**Figure 5A**). These results suggest that there were clonal expansions (i.e., increased clonality) in both the allografts and the circulating B cell pools in the first year post-Tx, but diversity eventually increased in the circulating blood clones and in the allograft in some cases during the late post-Tx period. Focusing on the 4 individuals with sequencing data whose clones were analyzed in all three time brackets, we compared how individual clones that spanned two consecutive time brackets changed in size (**Figure 5B**). We observed no clear trend in clone size changes between early and middle time points, whereas clones detected in the middle time bracket were significantly more likely to decrease in size in the late time bracket (**Figure 5B**), consistent with the increased diversity of B cell clones at this transitioning period shown in **Figure 5A**.

### B-cell clones found in pediatric blood and ileum allografts exhibit greater mixing than those in normal adults

**Figure 6:**
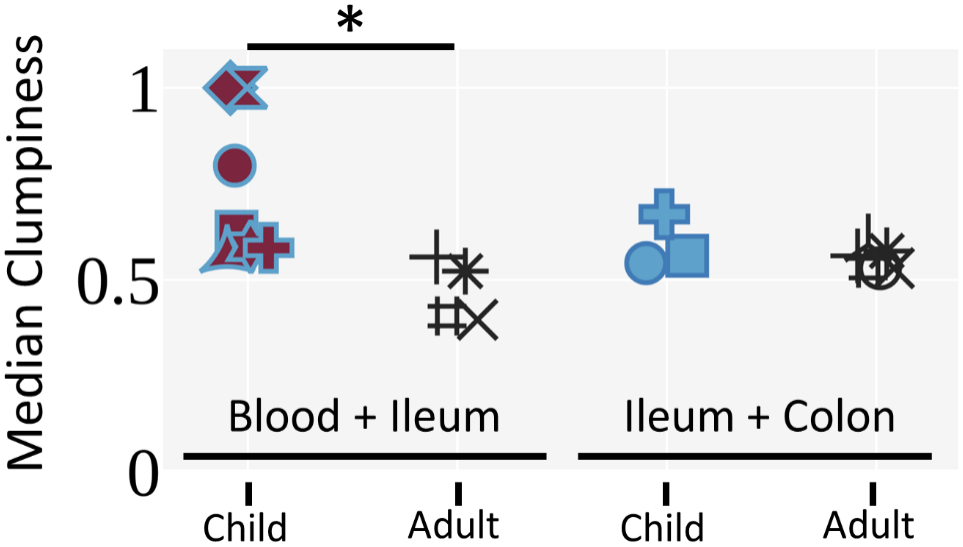
Pediatric ITx patients exhibit increased clumpiness between the blood and ileum allograft compared to deceased adult donors. Median clone clumpiness per individual for a given pair of tissues is shown. Only clones with 3 or more unique sequences that were sampled in both tissues were included. Medians were filtered for having greater than 5 clones. The Mann-Whitney U test was performed to determine statistical significance (*p<0.05). Individuals are marked as in **Figure 4**.

By comparing allograft and blood specimens after combining samples from all timepoints, we were able to evaluate the extent to which clones detected in two tissues mixed or segregated their unique sequences from each tissue within their lineages using a metric called clumpiness. Clumpiness is a metric that quantifies how mixed two metadata labels are within a hierarchical lineage – in our case, how mixed two tissues are within a clonal lineage (see Methods and **Supplementary Figure 5** for an extended explanation). We used clumpiness to quantify the extent to which mutations from different tissues within clonal lineages were shared along common branches (11, 49) (**Supplementary Figure 5**). Surprisingly, we observed significantly greater clumpiness between the post-Tx blood and ileum allograft in the pediatric transplant cohort compared to the low clumpiness that we had previously reported between the blood and ileum in healthy (no known immunity-related issues) deceased adult donors (11) (**Figure 6**). There was no significant difference in clumpiness between the ileum and colon of pediatric transplant recipients compared to normal adults (**Figure 6**). Given that the mutation levels of B cells in pediatric ITx patients’ gut samples tended to be lower than those in adult controls, we performed additional analyses to exclude the possibility that the higher clumpiness between blood and ileum in the pediatric transplant recipients was the result of a lack of complexity due to lack of mutations in the children’s B cell clones. We found that the clumpiness differences remained significant even when filtering for B cell clones that possess many mutations (>2% average V gene mutation) or contain a trunk of at least 5 shared mutations (**Supplementary Figure 6**). Lastly, the mixing between the blood and ileum allograft did not appear to change with time, but rather remained at an overall comparable level throughout a majority of time points in each patient (**Supplementary Figure 7**).

### Phenotypic and clonal overlap across intestinal allograft and native tissue with differential distribution patterns in large intestine vs small intestine

**Figure 7:**
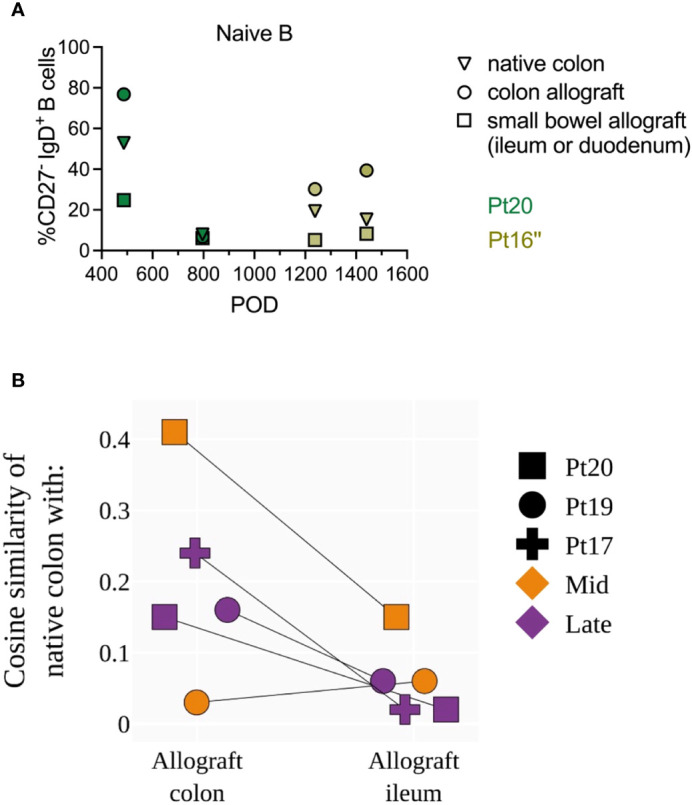
Phenotypic and repertoire analysis of recipient B cells in pan-scope biopsies of pediatric ITx recipients. **(A)** Percentages of recipient naive (CD27^-^IgD^+^) and memory B cells (CD27^+^ IgD^+/-^) in native colon, colon allograft and small bowel (ileum or duodenum) allograft during quiescence at late post-Tx time points (POD 400–1600) from two pediatric ITx patients. **(B)** Cosine similarities of BCR repertoires between native colon and colon allograft, and between native colon and ileum allograft for three pediatric ITx patients are shown at mid and/or late post-Tx time brackets (defined as in **Figure 1**). Due to the limited number of patients and variable number of time points available between patients for this analysis, we did not assess statistical significance but instead describe biologically informative trends.

When pan-scope samples were available from our ITx recipients, we compared recipient B cell phenotypes and repertoires among intestinal allografts (small bowel and colon) and non-transplanted native colon tissues collected at the same time point. Due to the limited number of patients and variable numbers of time points available for this analysis between patients, we only describe biologically informative trends here without assessing statistical significance (**Figure 7**). Colon allografts tended to show higher percentages of naïve B cells than ileum allografts at the same time in 3 out of 4 late post-Tx (POD>365) samples in the two young pediatric patients tested (**Figure 7A**). The percentages of naïve B cells in native colon were in general lower than those in colon allografts but greater than those in ileum allografts (**Figure 7A**). BCR-seq data on pan-scope samples collected at mid and late stages post-Tx in three young pediatric patients showed variable BCR clonal overlap between native colon and intestinal allograft samples, as reflected by cosine similarity values as high as 0.41 (**Figure 7B**). Cosine similarity indices were higher between native colon and colon allograft compared to those between native colon and ileum allograft (**Figure 7B**). This difference was not attributable to the lower number of clones in the ileum allografts than the colon allografts (**Supplementary Table S6**). Our data suggest ongoing B cell repertoire establishment in both the intestinal allograft and native mucosa after transplant, with differential clonal distribution patterns within the large intestine vs the small intestine.

## Discussion

The human body is inhabited by diverse and dynamic multitudes of B cells. Although the stimuli, regulation, networks, and cellular partnerships that shape these populations are increasingly well studied, how they attain homeostasis within a tissue domain remains unknown. Here, the analysis of serial surveillance biopsies following solid organ transplantation allowed analysis of recipient B cells immigrating into the allografted intestinal mucosa in immunosuppressed conditions in the absence of rejection, providing a relevant biological scenario that may mimic how B cell steady state is attained in human tissues. A study of human fetal BCR development revealed diverse repertoires with increasing IgH CDR3 lengths with advancing gestational age (14). To our knowledge, all previous intestinal B cell repertoire studies have included only cross-sectional data, as serial biopsies would be difficult to obtain within human individuals in settings other than transplantation. While the applicability of our results is potentially limited by the immunosuppressive therapy required in the transplant setting, our study is the first to provide longitudinal data on B cell dynamics in the human intestine. We found that recipient B cells rapidly repopulate the intestinal allograft, replacing over 80% of donor B cells within the first year post-Tx in most patients we studied. This is in contrast to T cell repopulation, where over 50% of donor CD4^+^ and CD8^+^ T cells are maintained more than 1 year in the allograft in the absence of significant rejection and with older donor age (≥1 year old), but where recipient repopulation is significantly more rapid in the presence of rejection or with infant donors (<1 year old) even in the absence of rejection (23, 25, 39). Our current study shows no correlation between the rate of recipient B cell entry into intestinal allografts and rejection. However, we did see an association of rapid recipient B cell repopulation and infant donor ileal allografts. This may be explained by the following possibilities: 1) the immature state of the infant mucosal immune system and immune cells in the gut (53–55), requiring increased population of the mucosal compartment by circulating B cells; and/or 2) the fact that younger donors (<2 years old tested in the cited study, including 4, 16, 18, and 21 months old) have more isolated lymphoid follicles in the small intestine than older individuals (53), resulting in increased numbers of anatomical niches for residence of recipient B cells immigrating from the circulation. The distribution pattern of B cells in the lamina propria of intestinal mucosa beyond the lymphoid follicles has not been reported for human infants. We hypothesize that this distribution may be patchy, in contrast to a more diffuse mucosal distribution pattern for T cells, on the basis of our observations from staining of CD20^+^ B cells and CD3^+^ T cells in intestinal mucosa during quiescence after ITx (30), and from our observations of fluctuating recipient B cell contributions to intestinal allograft biopsies taken from different sites at adjacent timepoints (**Supplementary Figure 1A**). In contrast, recipient T cell representation in these biopsies tends to be more stable at adjacent timepoints (23, 25, 39).

Donor selection for ITx depends on donor availability and other primary surgical and medical concerns, such as organ size, blood typing, and Model for End-Stage Liver Disease (MELD) score. However, an understanding of the role of donor age in immunological maturity of the graft, including not only B cells but also T cells, could greatly improve graft outcomes and post-Tx management. For instance, our published (25) and ongoing studies suggest that the combination of blood macrochimerism (≥4% donor T cells) status and T cell replacement rate in intestinal allografts in patients with donors older than 1 year old has the potential to serve as a biomarker of rejection. However, in patients with infant donors, rapid T cell replacement occurs in ileal allografts without rejection, suggesting that this putative biomarker applies only to allografts that have reached a certain level of immunological maturity at the time of transplant. Further studies of the spatial distribution of graft-infiltrating recipient T and B cells in patients with infant, young pediatric and adult donor grafts will provide further insight on this issue. On the other hand, GVHD is a relatively rare complication after ITx, but can be life-threatening. We have demonstrated that blood macrochimerism occurs frequently after ITx, without GVHD (22, 24, 25). The B cell phenotyping and repertoire studies described herein involved patients who did not develop GVHD, which has been largely absent from patients in our cohort, regardless of donor or recipient age.

The observed detection of DSA-secreting recipient B cells in the graft itself is consistent with previous reports from heart (56, 57), liver (58), lung (59) and kidney (60, 61) allografts, and suggests that local alloantibody production may contribute to intestinal allograft rejection, which is strongly associated with the development of DSA (26, 62). It is noteworthy that serum DSA negativity did not necessarily correlate with graft DSA negativity or vice versa in those studies. Serum DSA could be completely different than graft DSA, or only a subset of serum DSAs may be detected in the matched graft tissue (59). The latter might occur if serum DSAs have differential binding abilities to the allograft, and/or are present in the tissue at too low a concentration to bind to antigen present locally. On the other hand, there are several speculated mechanisms facilitating local DSA production. First, the formation of tertiary lymphoid organs (TLOs) in chronically rejected allografts creates a microenvironment of abundant alloantigen and self-antigens released upon tissue damage (63). The restricted lymphatic drainage features of these TLOs are believed to trap antigens and initiate a pronounced local immune response, likely leading to distinct local DSA production compared to lymphoid tissues. Second, differential gut mucosal and systemic microbiota exposures can also shape the B cell repertoire (4) and may affect their DSA production profile *in situ*.

Our analysis of specificity and antibody secretion by B cells derived from the local allograft was limited by low B cell availability due to small biopsy samples and by poor survival of graft-derived B cells for study. Although we attempted multiple cell culture protocols (40, 41), graft-derived B cells did not survive well or secrete sufficient antibody in cultures of mucosal fragments or cell suspension cultures after sorting. This is consistent with observations that intestinal IgG-secreting cells rapidly cease antibody secretion in culture (64). Further investigation using a greater amount of mucosal tissue obtained from stoma revision, closure, or graft explant surgeries will be needed to strengthen this finding. Experimental optimization, such as utilization of Secretome media, addition of a proliferation-inducing ligand (APRIL), and hypoxic conditions, are expected to improve the survival of graft-derived B cells in *in vitro* functional analyses (65–67).

Increased levels of donor B cell chimerism were detected in the peripheral blood of MVTx compared to iITx recipients, confirming previous findings (22, 25). Both donor and recipient circulating B cells were dominated by naïve phenotypes over the entire follow up period up to 1000 days post-Tx. However, in contrast to infant donor intestinal grafts, the donor B cells from older (≥1 year old) intestinal grafts were predominated by memory cells. Recipient B cells entering the intestinal allograft took on the memory phenotype, including BRM phenotypes that are barely detectable in the circulation, consistent with their gut-resident memory identity. Recipient memory B cells surprisingly maintained relatively constant frequencies (20–50%) in the mid through late post-Tx periods. In contrast to chimerism, where allografts from younger donors had faster recipient B cell replacement, there was no relationship between the naïve vs memory subset of recipient B cell frequencies in the allograft and donor age.

High throughput BCR sequencing data provided further insights into repertoire establishment in the intestinal graft over time during periods of quiescence. First, older age was associated with higher B cell mutation levels in both blood and ileum. Furthermore, B cells in the ileum showed higher mutation levels compared to those in the blood, both in pediatric ITx patients and adult deceased donor controls, similar to our earlier observations in adult organ donors (11). Finally, we detected greater trafficking of recipient B cells between the blood and the ileum allograft in pediatric ITx patients compared to adult non-transplanted controls (11), as demonstrated by the clumpiness measure in clonal lineages that overlapped between the blood and the ileum. While the level of trafficking remained stable over time, we tended to observe reduced evenness and diversity of the mucosal B cell repertoire in the first 3 months compared to pre-Tx blood and allograft and from 3 months to 1 year post-Tx, with some allografts reverting to pre-Tx high diversity levels in the late post-Tx period. Mutation levels in variable regions of B cells residing in allografts rose initially post-Tx and tended to be greater than those in the circulation, but they did not show continuous increases over time. Our findings are consistent with the possibility that a considerable fraction of B cell clones traffic continuously between the blood and the ileum allograft, rather than migrating from the blood to the ileum allograft (or vice versa) and then remaining there. While an initial surge of recipient B cell clones enters the ileum allograft in the early and middle time brackets and expand, the more uniform and evenly high diversity in the late time bracket may be explained by the possibility that many years of trafficking between the blood and ileum allograft are required post-Tx before homeostasis is achieved. Pan-scope biopsy analysis from pediatric recipients late post-Tx demonstrated a BCR clonal overlap between native colon and intestinal allografts with potentially differential distribution patterns in large intestine vs small intestine. Taken together, data in both the temporal and spatial dimensions provides new insights into the achievement of immune homeostasis in native and allograft intestinal mucosa.

From these data we conclude that the pediatric intestinal allograft serves as an open space for recipient B cell clones to enter, and then expand and mutate. Even after 1 year, however, recipient B cell clones in the allograft revert to mutation and diversity levels comparable to what is observed early post-Tx. While we cannot rule out the possibility that recipient B cell clonal expansion within the allograft represents an immunological attack against the allograft, this seems unlikely since our repertoire analysis only included samples lacking evidence for rejection. Rather, we hypothesize that the observed recipient B-cell repertoires reflect continuous entry and turnover from the circulation into lymphoid follicle-enriched gut tissue from pediatric donors (53). It is possible that this dynamic process echoes what occurs normally through human childhood and that steady state had not yet been established by the end of the follow-up period. Previous analyses demonstrated that the intestinal organs and lymph nodes contained a separate network of B cell clones from those in the blood, spleen and bone marrow (11), but these analyses were performed on tissues from adult deceased donors. The differences observed here may reflect primarily the young age of our donors and recipients. If so, our data may provide novel insight into the dynamic establishment of the human intestinal mucosal B cell repertoire during childhood. If, on the other hand, our results are particular to the intestinal allograft setting with long-term immunosuppression and do not reflect normal developmental processes, then they suggest an important failure to establish normal immune homeostasis following ITx. Studies following these patients for even longer periods, well into adulthood, will be needed to distinguish between these possibilities.

We have considered the possibility that ATG, as T-cell-depleting induction therapy, or tacrolimus and steroids as maintenance immunosuppression, might have a direct impact on B cells in the allograft. It is possible that the depletion of circulating and, to a lesser extent, graft T cells by ATG induction might open up physical space for recipient B cell repopulation and interactions between T cells and B cells in intestinal graft mucosa, including gut-associated lymphoid follicles. B cell depleting therapy such as rituximab during DSA+ mixed rejection treatment is expected to reduce the absolute number of circulating, and to a lesser extent, graft B cells. The exact effect of such B cell depleting therapy on B cell dynamics and repertoire evolution will need to be further investigated on expanded cohorts with sufficient ileal biopsy samples from no rejection, TCMR, and mixed rejection time points. Future studies could use rodent (68–70) and pig (71) ITx models to disentangle the effects of immunosuppression from natural immunological reconstitution processes.

Further phenotypic, repertoire and functional studies are needed to explore the local and systemic B cell response against both alloantigens and microbes to understand their contributions to graft outcomes. We recently described the dynamic establishment of tissue resident memory T cell repertoires, their crosstalk with the circulating T cell pool, and their antigen recognition patterns after human ITx (39). Strategies to control alloreactive B and T cells while maintaining anti-microbial B and T cells are desired to overcome rejection and induce tolerance, while preventing infection after transplantation. Further analyses to determine the possible role of Bregs, including those expressing TIM-1 and IL-10 and transitional B cells might provide biomarkers or predictors of graft rejection (33–35). Further research is needed in larger cohorts with broader donor and recipient age ranges, longer-term follow up, and larger sampling of B cell dynamics, repertoire, and functional studies locally and systemically.

## Data availability statement

The datasets presented in this study can be found in online repositories. The names of the repository/repositories and accession number(s) can be found below: PRJNA1031101 (SRA).

## Ethics statement

The studies involving humans were approved by Columbia University Institutional Review Board. The studies were conducted in accordance with the local legislation and institutional requirements. Written informed consent for participation in this study was provided by the participants’ legal guardians/next of kin.

## Author contributions

JF: Conceptualization, Investigation, Methodology, Project administration, Resources, Supervision, Writing – original draft, Writing – review & editing, Data curation, Formal analysis, Validation, Visualization. TH: Conceptualization, Data curation, Formal analysis, Investigation, Methodology, Validation, Visualization, Writing – original draft, Writing – review & editing, Software. EW: Conceptualization, Data curation, Formal analysis, Investigation, Methodology, Validation, Visualization, Writing – original draft, Writing – review & editing, Supervision. WM: Data curation, Investigation, Methodology, Software, Writing – review & editing. KL: Data curation, Investigation, Methodology, Writing – review & editing. KF: Data curation, Investigation, Writing – review & editing. RJ: Data curation, Investigation, Writing – review & editing. AGo: Data curation, Investigation, Writing – review & editing. AS: Data curation, Investigation, Writing – review & editing. ML: Investigation, Writing – review & editing. CM: Data curation, Investigation, Project administration, Resources, Writing – review & editing. KR: Project administration, Resources, Writing – review & editing, Data curation, Investigation. WJ: Data curation, Investigation, Writing – review & editing. MV: Project administration, Resources, Writing – review & editing. RM: Investigation, Resources, Writing – review & editing. MK: Investigation, Resources, Writing – review & editing. SW: Investigation, Resources, Writing – review & editing. ND: Investigation, Project administration, Supervision, Writing – review & editing. SR: Investigation, Resources, Writing – review & editing. AI: Formal analysis, Investigation, Writing – review & editing. E-RV: Formal analysis, Investigation, Writing – review & editing. AGr: Resources, Writing – review & editing. JW: Investigation, Resources, Writing – review & editing. DF: Data curation, Funding acquisition, Investigation, Project administration, Resources, Supervision, Writing – review & editing. EL: Data curation, Formal analysis, Investigation, Methodology, Resources, Software, Supervision, Validation, Visualization, Writing – review & editing. MM: Investigation, Resources, Supervision, Writing – review & editing. TK: Investigation, Resources, Supervision, Writing – review & editing. UH: Conceptualization, Data curation, Formal analysis, Funding acquisition, Investigation, Methodology, Project administration, Resources, Software, Supervision, Validation, Visualization, Writing – original draft, Writing – review & editing. MS: Conceptualization, Formal analysis, Funding acquisition, Investigation, Methodology, Project administration, Resources, Supervision, Writing – original draft, Writing – review & editing.

## Supplementary material

Supplementary Table 1 Epidemiological and clinical characteristics of ITx patients. Those with B cell chimerism, phenotypic and sequencing data include Pts 4 reTx, 14, 16, 16 reTx, 17, 19, 20, 21, 21 reTx, 22, 23, 24, 25, 26, 27. SBS: Short Bowel Syndrome. NEC, Necrotizing enterocolitis; TCMR, T-cell mediated rejection; DSA, Donor-Specific Antibody; PTLD, Posttransplant lymphoproliferative disease.

Supplementary Table 2 Epidemiological and clinical characteristics of adult healthy control deceased organ donors.

Supplementary Table 3 HLA class I typing and anti-HLA allele group antibodies used to distinguish donor from recipient cells in ITx recipients. HLA-A09 is a broad antigen HLA-A serotype that recognized the HLA-A23 and HLA-A24 serotypes. HLA-A28 is a broad antigen HLA-A serotype that recognized the HLA-A68 and HLA-A69 serotypes. HLA-B12 is a broad antigen HLA-B serotype that recognized the HLA-B44 and HLA-B45 serotypes. Each selected antibodies have been tested on pre-Tx donor and recipient PBMCs for their specificity to recognize donor, but not recipient, cells, or vice versa.

Supplementary Table 4 Sequencing stats per subject. The stats shown pertain only to the tissues that are listed. In the case that an individual has other tissues not listed here, the stats for those unlisted tissues are not included. As mentioned in the Methods section, samples with rejection were not used in the analyses unless specifically stated in the figure.

Supplementary Table 5 Number of samples per POD bracket by tissue. POD brackets: P, Pre-transplant (POD0); E, Early (PODs 1–90); M, Mid (PODs 91–365); L, Late (PODs >365).

Supplementary Table 6 Number of clones used in the cosine similarity analysis in **Figure 7B**. Mid (PODs 91–365); Late (PODs >365).

Supplementary Figure 1 Gating strategy for phenotyping B cell subsets in Pt20 POD73 PBMC **(A)** and POD74 ileal allograft biopsy **(B)** samples. HLA-specific markers distinguishing transplant donor (blue) and recipient (red) are used to identify transplant-originating donor cells in PBMC and allograft mucosa. Gates and % shown are only for recipient cells (red). Memory B cells: CD27^+^IgD^+/-^. Naïve B cells: CD27^-^IgD^+^. Transitional B cells: CD24^+^CD38^+^ among naïve B cell gating. Mature Naïve B cells: CD24^-/dim^CD38^-/dim^ among naïve B cell gating. ASC (antibody secreting cells): CD24^-^CD38^+^ among memory B cell gating. CD24^+^ Memory: CD24^+^CD38^-/dim^ among memory B cell gating. BRM: CD69^+^CD45RB^+^ B cells.

Supplementary Figure 2 Normalized area under the curve (AUC) values of recipient B cell chimerism in allograft in patients **(A)** with or without acute cellular rejection (ACR) and **(B)** with or without *de novo* Class I/II DSA in serum during early post-Tx period (up to POD90). Dashed lines from the bottom to the top within each condition represent the first quartile, median, and third quartile of data distribution, respectively. No significant difference was detected by Mann-Whitney U test (p>0.05).

Supplementary Figure 3
**(A)** *De novo* development of Class I (upper panel) and Class II (lower panel) DSA detected in post-Tx serum correlates with higher rates of moderate or severe ACR. **(B)** Local production of DSA by mucosal recipient B cells matched the DSA specificity detected in the serum on the same day in a patient during graft explant (Pt14 POD1764) due to chronic rejection with previously persistent ACR.

Supplementary Figure 4 The fraction of clones per sample with an average v gene mutation frequency >2% by POD is shown in peripheral blood and ileum allograft. The median fraction of mutated clones among adult deceased donors is shown by the dashed lines. Individuals are marked as in **Figure 4**. Green markers indicate pre-Tx samples (samples taken at POD0).

Supplementary Figure 5 Clumpiness is a metric quantifying the extent to which specific metadata labels are clumped together in branches of a lineage. Clumpiness values range from 0 to 1. A clumpiness value of 1 between two labels means they are perfectly mixed within a lineage (always found together on all of the branches of a lineage), while a clumpiness value of 0 means the two labels are always on separate branches. Likewise, a clumpiness value of 0.5 means the labels are somewhat mixed within the lineage (the two labels are found together on some of the same branches, but are also found on other branches without the other label).

Supplementary Figure 6 Pediatric transplant patients exhibit increased trafficking between the blood and ileum allograft among mutated **(A)** and trunk **(B)** clones compared to adult deceased donor controls. The median clumpiness per individual for a given pair of tissues is shown. In panel **(A)**, clones were filtered for having 3 or more sequenced nodes in their lineages, being mutated (average gene mutation frequency >2%), and being sampled in both tissues that were compared. In panel **(B)**, clones were filtered for having 3 or more sequenced nodes, having a trunk (defined as having at least 5 shared mutations in 85% of a lineage’s sequenced nodes; see Methods), and being sampled in both tissues that were compared. Only medians with greater than 5 clones were included. The Mann-Whitney U test was performed to determine statistical significance (*p<0.05). Individuals are marked as in **Figure 4**.

Supplementary Figure 7 Median clumpiness between the blood and ileum allograft (or pre-Tx ileum for POD0) per individual by POD was shown. Clones were filtered for having 3 or more unique sequences, and being sampled in both tissues that were compared. Only medians with greater than 5 clones were included. Median clumpiness between the blood and ileum tissues in adult deceased donors is shown by the dotted dashed line. Individuals are marked as in **Figure 4**.
