# Supplementary material for: Dynamic establishment and maintenance of the human intestinal B cell population and repertoire following transplantation in a pediatric-dominated cohort
Source: Front Immunol. 2024 Jun 28;15:1375486. doi: 10.3389/fimmu.2024.1375486 (PMC11239347; doi:10.3389/fimmu.2024.1375486)
Supplement: Supplementary Table 1 — Epidemiological and clinical characteristics of ITx patients. Those with B cell chimerism, phenotypic and sequencing data include Pts 4 reTx, 14, 16, 16 reTx, 17, 19, 20, 21, 21 reTx, 22, 23, 24, 25, 26, 27. SBS: Short Bowel Syndrome. NEC, Necrotizing enterocolitis; TCMR, T-cell mediated rejection; DSA, Donor-Specific Antibody; PTLD, Posttransplant lymphoproliferative disease. [file DataSheet_1.docx]

**Table S1. Epidemiological and clinical characteristics of ITx patients.** Those with B cell chimerism, phenotypic and sequencing data include Pts 4 reTx, 14, 16, 16 reTx, 17, 19, 20, 21, 21 reTx, 22, 23, 24, 25, 26, 27. SBS: Short Bowel Syndrome. NEC: Necrotizing enterocolitis. TCMR: T-cell mediated rejection. DSA: Donor-Specific Antibody PTLD: Posttransplant lymphoproliferative disease.

| Pt (#) | Indication for Transplant | Tx Type | Recipient Age at Tx (years) | Recipient Sex | Donor Age (years) | Donor Sex | Rejection (intestinal mucosal biopsy) | GVHD | Death  or Graft Removal | *De Novo* DSA (Serum) |
| --- | --- | --- | --- | --- | --- | --- | --- | --- | --- | --- |
| 1 | Desmoid; Gardner's Syndrome | MVTx | 53 | Male | 6 | Male | Early-Mid-Late TCMR (mild) | No | No | No |
| 2 | Volvulus; SBS | iITx | 47 | Female | 48 | Female | Early TCMR (moderate) | No | Death (Sudden death) | No |
| 3 | Pseudo-obstruction | MVTx | 17 | Female | 21 | Male | Early-Mid-Late TCMR (mild) | No | Death (PTLD) | No |
| 4 | Pseudo-obstruction; Berdon syndrome | iITx | 6 | Female | 2 | Male | Early mixed Rejection (mild, moderate to severe); Mid TCMR (mild to moderate); Late TCMR (mild to moderate, moderate to severe) | No | Graft Removal | Early  Class I, MFI <6,000,  Class II MFI <6,000  Late  Class I, MFI <6,000,  Class II MFI <6,000 |
| 4  reTx | Graft loss | MVTx | 14 | Female | 4 | Male | Mid TCMR (mild) | No | No | No |
| 5 | Desmoid; SBS | iITx | 27 | Male | 13 | Female | Early TCMR (mild) | No | No | No |
| 6 | Volvulus; SBS | iITx | 3 | Male | 3 | Female | Early TCMR (mild); Late TCMR (mild to moderate) | Self-limited (skin) | No | *Preformed  Class II, MFI <6,000 |
| 7 | Entire porto-mesenteric  system thrombosis | MVTx | 53 | Male | 44 | Male | Early TCMR (mild); Mid TCMR (mild to moderate) | No | Death (Pneumonia) | No |
| 9 | NEC; SBS | iITx | 2 | Male | 1 | Female | Early mixed Rejection (moderate); Mid-Late TCMR (mild) | No | Death (respiratory failure) | Early  Class I, MFI <10,000,  Class II MFI <6,000 |
| 10 | Extensive porto-mesenteric  system thrombosis; SBS | MVTx | 32 | Female | 2 | Male | Early mixed Rejection (moderate); Mid (moderate to severe); Late (moderate) | No | Death (sepsis after severe rejection) | *Preformed  Class I/II, MFI >10,000; Early/Mid  Class I, MFI <10,000,  Class II MFI <10,000; Late  Class II MFI <10,000 |
| 13 | Tufting enteropathy | iITx | 2 | Male | 2 | Male | Early TCMR (moderate); Mid-Late TCMR (mild) | No | Graft Removal | No |
| 14 | Tufting Enteropathy | iITx | 2 | Male | <1 | Female | Early mixed rejection (mild);  Mid-Late mixed rejection (Moderate) | No | Graft Removal | Early  Class I, MFI >10,000,  Class II MFI <6,000  Late  Class I, MFI <6,000,  Class II MFI >10,000 |
| 15 | Microvillus inclusion disease | MVTx | 5 | Female | 7 | Female | Mid-Late TCMR (mild to moderate) | No | No | No |
| 16 | NEC; SBS | LITx | 1 | Male | <1 | Male | Early TCMR (moderate to severe) | No | Graft Removal | No |
| 16 reTx | Graft loss | MVTx | 3 | Male | 1 | Male | Early TCMR  (mild to moderate);  Mid-Late TCMR (mild) | No | No | No |
| 17 | Tufting Enteropathy | iITx | 5 | Male | 5 | Male | Early mixed rejection (mild to moderate)  Mid-Late TCMR (mild-moderate) | No | No | Early  Class II MFI >6,000 |
| 18 | Necrotic volvulus and TPN associated liver disease | MVTx | 1 | Male | <1 | Female | No | No | No | Early  Class II MFI <6,000 |
| 19 | SBS secondary to *in utero* volvulus | MVTx | 3 | Male | <1 | Female | No | No | No | Early  Class II MFI <10,000 |
| 20 | SBS secondary to malrotation/volvulus | iITx | 1 | Female | <1 | Female | Early mixed Rejection  (mild to moderate); Late TCMR (mild, severe) | No | No | Early  Class I, MFI <10,000,  Class II MFI <6,000 |
| 21 | Microvillus Inclusion | MVTx | 2 | Female | <1 | Male | Late TCMR (mild, moderate to severe) | No | Graft Removal | Late  Class II MFI <6,000 |
| 21 reTx | Graft loss | MVTx | 5 | Female | <1 | Female | Early-Mid TCMR (mild) | No | No | No |
| 22 | Budd Chiari Syndrome | MVTx | 44 | Female | 23 | Male | Early TCMR (mild) | No | Death (Sepsis) | *Preformed  Class I, MFI <6,000 |
| 23 | SBS, midgut atresia | MVTx | 2 | Male | <1 | Male | Early TCMR  (mild to moderate)  Late TCMR  (moderate to severe) | No | Death (Viral infection) | *Preformed  Class I, MFI <6,000,  Class II MFI <10,000 |
| 24 | SBS, cholestasis, SVC thrombosis | iITx | 6 | Male | 1 | Male | Early mixed Rejection (mild to severe) | No | Graft removal | Early  Class I, MFI <10,000,  Class II MFI >10,000 |
| 25 | SBS, biliary stricture, portomesenteric thrombosis | MVTx | 9 | Male | 3 | Female | Early mixed Rejection (mild)  Mid mixed Rejection (mild-moderate) | No | No | Early  Class I, MFI <10,000,  Class II MFI >10,000 |
| 26 | HTN, SBS secondary to midgut volvulus and mesenteric ischemia | iITx | 41 | Female | 26 | Female | Early-Mid TCMR  (mild to moderate) | No | No | Early  Class II, MFI >2,000 |
| 27 | SBS secondary to NEC | MVTx | 3 | Female | 3 | Female | Early-Mid-Late TCMR (mild) | No | No | No |

**Table S2. Epidemiological and clinical characteristics of adult healthy control deceased organ donors.**

| Donor (#) | Age (years) | Sex | Cause of Death | Tissue Usage |
| --- | --- | --- | --- | --- |
| 145 | 58 | Male | Cerebrovascular accident | PBMC, ileum; BCR sequencing |
| 149 | 55 | Male | Anoxia | PBMC, ileum; BCR sequencing |
| 168 | 56 | Female | Cerebrovascular accident | PBMC, ileum; BCR sequencing |
| 181 | 46 | Male | Cerebrovascular accident | PBMC, ileum; BCR sequencing |
| 182 | 46 | Male | Cerebrovascular accident | PBMC, ileum; BCR sequencing |
| 207 | 23 | Male | Head Trauma | PBMC, ileum; BCR sequencing |
| 425 | 30 | Female | Anoxia | PBMC, ileum; B cell phenotyping |
| 430 | 18 | Male | Brain Hemorrhage | PBMC, ileum; B cell phenotyping |
| 442 | 19 | Male | Motor Vehicle Accident | PBMC, ileum; B cell phenotyping |
| 530 | 28 | Male | Cerebrovascular/stroke | PBMC, ileum; B cell phenotyping |
| 531 | 46 | Male | Cerebrovascular/stroke | PBMC, ileum; B cell phenotyping |

**Table S3. HLA class I typing and anti-HLA allele group antibodies used to distinguish donor from recipient cells in ITx recipients.** HLA-A09 is a broad antigen HLA-A serotype that recognized the HLA-A23 and HLA-A24 serotypes. HLA-A28 is a broad antigen HLA-A serotype that recognized the HLA-A68 and HLA-A69 serotypes. HLA-B12 is a broad antigen HLA-B serotype that recognized the HLA-B44 and HLA-B45 serotypes. Each selected antibodies have been tested on pre-Tx donor and recipient PBMCs for their specificity to recognize donor, but not recipient, cells, or vice versa.

| Pt (#) | Recipient  HLA type | Donor  HLA type | HLA class I allele group-specific mAbs  used to discriminate recipient from donor cells |
| --- | --- | --- | --- |
| 4 reTx | **A02** / A30  B42 / B53 | A68 / A74  B72 / B42 | Anti-HLA A02 (BB7.2) FITC  Anti-HLA A03 APC (First donor) |
| 14 | **A23** / A-  B50 / B- | **A03** / A68  B35 / B58 | Anti-HLA A09 Biotin  Anti-HLA A03 APC |
| 16 re Tx | A02 / A34  B15 / **B44** | **A24** / 29  B35 / **B44** | Anti-HLA B12 FITC (First donor B12^-^A9^-^)  Anti-HLA A09 Biotin |
| 17 | A30 / A31  B40 / B53 | A24 / A32  **B27** / B35 | Anti-HLA B27 FITC |
| 19 | A02 / A68  B39 / B48 | A02 / **A03**  B53 / B72 | Anti-HLA A03 APC |
| 20 | A02 / **A03**  B50 / B52 | A02 / A68  **B44** / B53 | Anti-HLA A03 APC  Anti-HLA B12 FITC |
| 21 | A01 / A11  B39 / B58 | **A03** / **A03**  B65 / B35 | Anti-HLA A03 APC |
| 21 reTx | A01 / A11  B39 / B58 | **A02** / A31  B18 / B60 | Anti-HLA A02/A28 FITC  Anti-HLA A03 APC (First donor) |
| 22 | A01 / A30  B15 / B53 | A29 / A34  B27 / **B45** | Anti-HLA B12 FITC |
| 23 | **A02** / A30  B15 / B57 | **A03** / A24  B35 / B44 | Anti-HLA A03 APC  Anti-HLA A02/28 Biotin |
| 24 | A01 / A11  **B08** / B41 | **A68** / A74  B07 /B57 | Anti-HLA B08 FITC  Anti-HLA A02/28 Biotin |
| 25 | **A02** / A03  B71 / B49 | A01 / A24  B08 / **B44** | Anti-HLA A02/28 Biotin  Anti-HLA B12 FITC |
| 26 | A01 / A33  B65 / B38 | A25 / A26  **B08** / B38 | Anti-HLA B08 FITC |
| 27 | A32 / A68  B35 / B57 | **A02** / **A03**  B07 / B51 | Anti-HLA A02 (BB7.2) FITC  Anti-HLA A03 APC |

**Table S4. Sequencing stats per subject.** The stats shown pertain only to the tissues that are listed. In the case that an individual has other tissues not listed here, the stats for those unlisted tissues are not included. As mentioned in the Methods section, samples with rejection were not used in the analyses unless specifically stated in the figure.

| Patient | Tissue | Unique sequences total | Copy number total | Number of samples without rejection | Number of samples with rejection | Number of PODs | Number of functional clones |
| --- | --- | --- | --- | --- | --- | --- | --- |
| Pt19 | Colon, Colon_allograft, Ileum_allograft, PBMC | 379,629 | 2,542,206 | 53 | 0 | 13 | 165,818 |
| Pt21 | Colon, Ileum, Ileum_allograft, PBMC | 265,522 | 1,356,710 | 47 | 1 | 11 | 148,305 |
| Pt23 | Colon, Ileum, Ileum_allograft, PBMC | 163,847 | 789,294 | 27 | 10 | 7 | 98,546 |
| Pt14 | Ileum_allograft, PBMC | 106,931 | 420,721 | 14 | 12 | 4 | 53,919 |
| Pt20 | Colon, Colon_allograft, Ileum_allograft, PBMC | 582,089 | 2,979,353 | 59 | 7 | 14 | 238,977 |
| Pt17 | Colon, Colon_allograft, Ileum_allograft, PBMC | 110,421 | 731,973 | 16 | 0 | 2 | 35,612 |
| Pt25 | Ileum_allograft, PBMC | 50,671 | 112,168 | 10 | 0 | 5 | 17,139 |
| D145 | Colon, Ileum, PBMC | 51,848 | 532,264 | 18 | 0 | N/A | 10,647 |
| D149 | Colon, Ileum, PBMC | 30,679 | 615,760 | 15 | 0 | N/A | 3,955 |
| D168 | Colon, Ileum, PBMC | 29,299 | 412,403 | 15 | 0 | N/A | 5,605 |
| D181 | Colon, Ileum, PBMC | 232,093 | 3,369,560 | 45 | 0 | N/A | 55,624 |
| D182 | Colon, Ileum, PBMC | 26,339 | 398,513 | 18 | 0 | N/A | 3,291 |
| D207 | Colon, Ileum, PBMC | 604,540 | 10,518,018 | 93 | 0 | N/A | 126,193 |

**Table S5. Number of samples per POD bracket by tissue.** POD brackets: P, Pre-transplant (POD0); E, Early (PODs 1-90); M, Mid (PODs 91-365); L, Late (PODs >365).

|  | PBMC | | | | Colon | | | | Colon allograft | | | | Ileum | | | | Ileum allograft | | | |
| --- | --- | --- | --- | --- | --- | --- | --- | --- | --- | --- | --- | --- | --- | --- | --- | --- | --- | --- | --- | --- |
| Patient | P | E | M | L | P | E | M | L | P | E | M | L | P | E | M | L | P | E | M | L |
| Pt19 | 3 | 6 | 6 | 4 | 0 | 0 | 2 | 4 | 0 | 0 | 4 | 4 | 0 | 0 | 0 | 0 | 0 | 12 | 6 | 2 |
| Pt21 | 2 | 2 | 4 | 14 | 0 | 0 | 0 | 2 | 0 | 0 | 0 | 0 | 8 | 0 | 0 | 0 | 0 | 4 | 6 | 5 |
| Pt23 | 6 | 4 | 2 | 2 | 0 | 0 | 2 | 0 | 0 | 0 | 0 | 0 | 3 | 0 | 0 | 0 | 0 | 4 | 2 | 2 |
| Pt14 | 4 | 0 | 0 | 2 | 0 | 0 | 0 | 0 | 0 | 0 | 0 | 0 | 0 | 0 | 0 | 0 | 0 | 0 | 4 | 4 |
| Pt20 | 6 | 2 | 8 | 14 | 0 | 0 | 2 | 2 | 0 | 0 | 2 | 2 | 0 | 0 | 0 | 0 | 0 | 2 | 8 | 11 |
| Pt17 | 0 | 0 | 0 | 2 | 0 | 0 | 0 | 4 | 0 | 0 | 0 | 5 | 0 | 0 | 0 | 0 | 0 | 0 | 0 | 5 |
| Pt25 | 2 | 6 | 0 | 0 | 0 | 0 | 0 | 0 | 0 | 0 | 0 | 0 | 0 | 0 | 0 | 0 | 0 | 0 | 2 | 0 |

**Table S6. Number of clones used in the cosine similarity analysis in Figure 7B.** Mid (PODs 91-365); Late (PODs >365).

| Patient | Mid  native colon | Late  native colon | Mid  colon allograft | Late  colon allograft | Mid  ileum allograft | Late  ileum allograft |
| --- | --- | --- | --- | --- | --- | --- |
| Pt20 | 471 | 5,019 | 1,629 | 10,859 | 7,159 | 23,187 |
| Pt19 | 1,786 | 18,386 | 25,267 | 8,064 | 10,804 | 11,232 |
| Pt17 | N/A | 2,839 | N/A | 6,578 | N/A | 16,981 |

**Supplemental Figure 1** Gating strategy for phenotyping B cell subsets in Pt20 POD73 PBMC (A) and POD74 ileal allograft biopsy (B) samples. HLA-specific markers distinguishing transplant donor (blue) and recipient (red) are used to identify transplant-originating donor cells in PBMC and allograft mucosa. Gates and % shown are only for recipient cells (red). Memory B cells: CD27^+^IgD^+/-^. Naïve B cells: CD27^-^IgD^+^. Transitional B cells: CD24^+^CD38^+^ among naïve B cell gating. Mature Naïve B cells: CD24^-/dim^CD38^-/dim^ among naïve B cell gating. ASC (antibody secreting cells): CD24^-^CD38^+^ among memory B cell gating. CD24^+^ Memory: CD24^+^CD38^-/dim^ among memory B cell gating. BRM: CD69^+^CD45RB^+^ B cells.


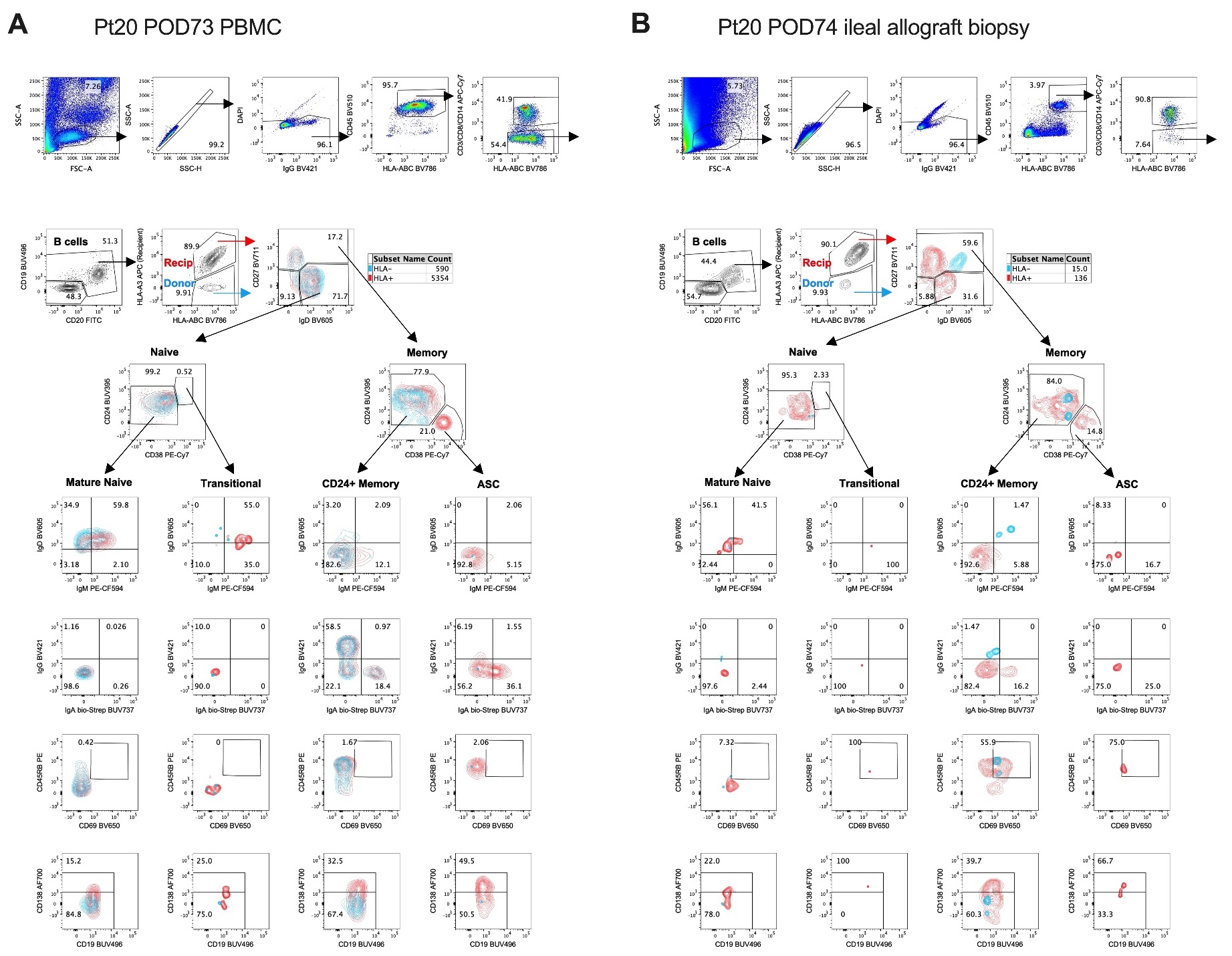


**Supplemental Figure 2** Normalized area under the curve (AUC) values of recipient B cell chimerism in allograft in patients (A) with or without acute cellular rejection (ACR) and (B) with or without *de novo* Class I/II DSA in serum during early post-Tx period (up to POD90). Dashed lines from the bottom to the top within each condition represent the first quartile, median, and third quartile of data distribution, respectively. No significant difference was detected by Mann-Whitney U test (p>0.05).


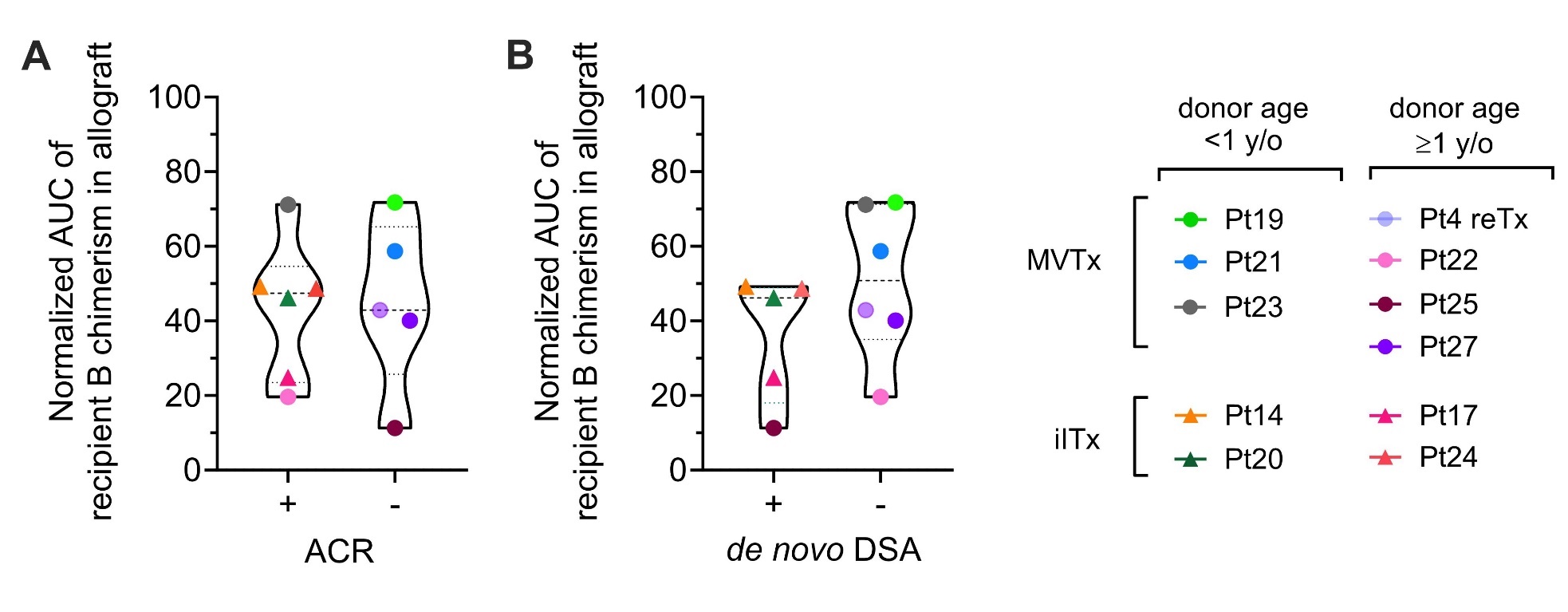


**Supplemental Figure 3** (A) *De novo* development of Class I (upper panel) and Class II (lower panel) DSA detected in post-Tx serum correlates with higher rates of moderate or severe ACR. (B) Local production of DSA by mucosal recipient B cells matched the DSA specificity detected in the serum on the same day in a patient during graft explant (Pt14 POD1764) due to chronic rejection with previously persistent ACR.


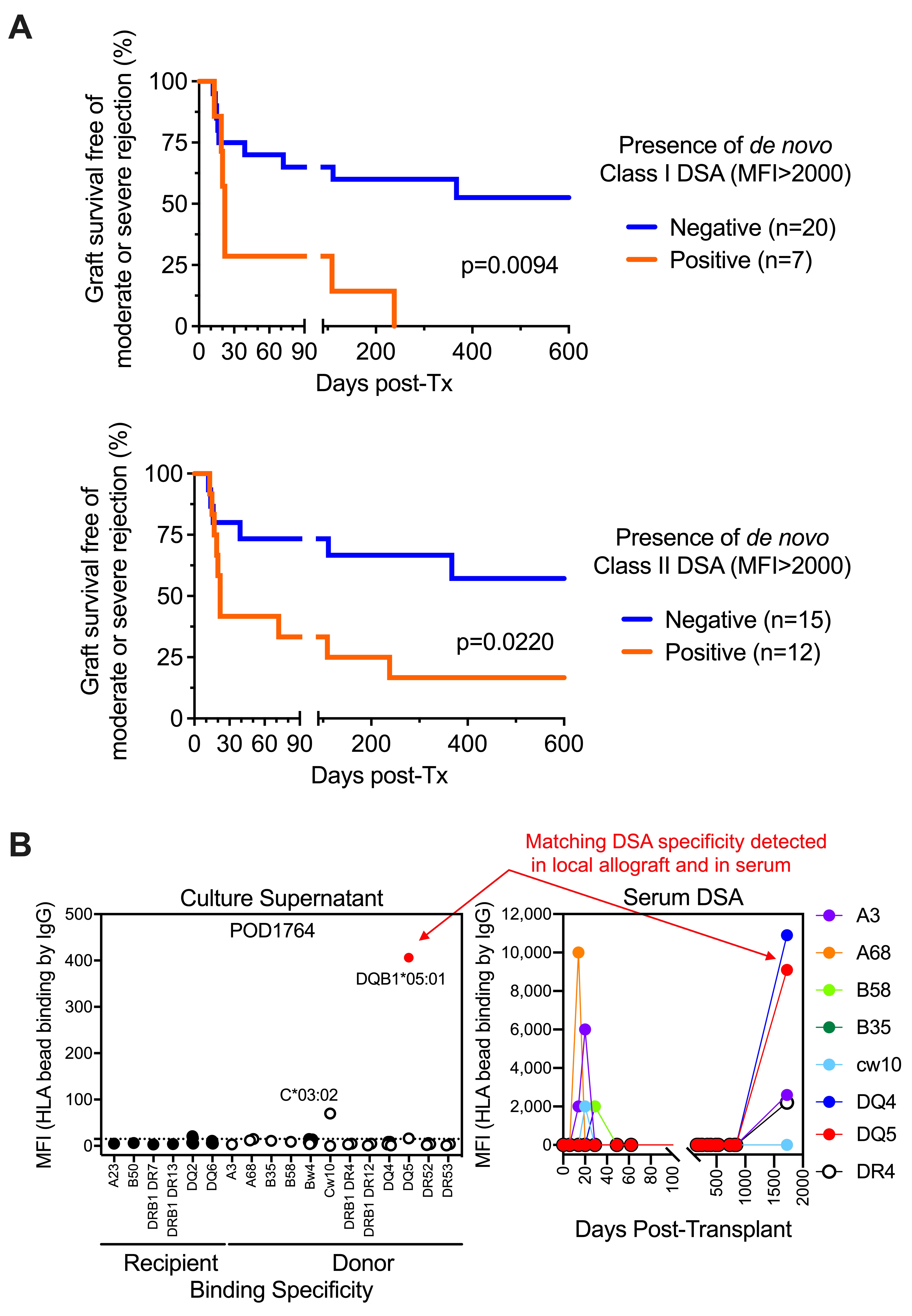

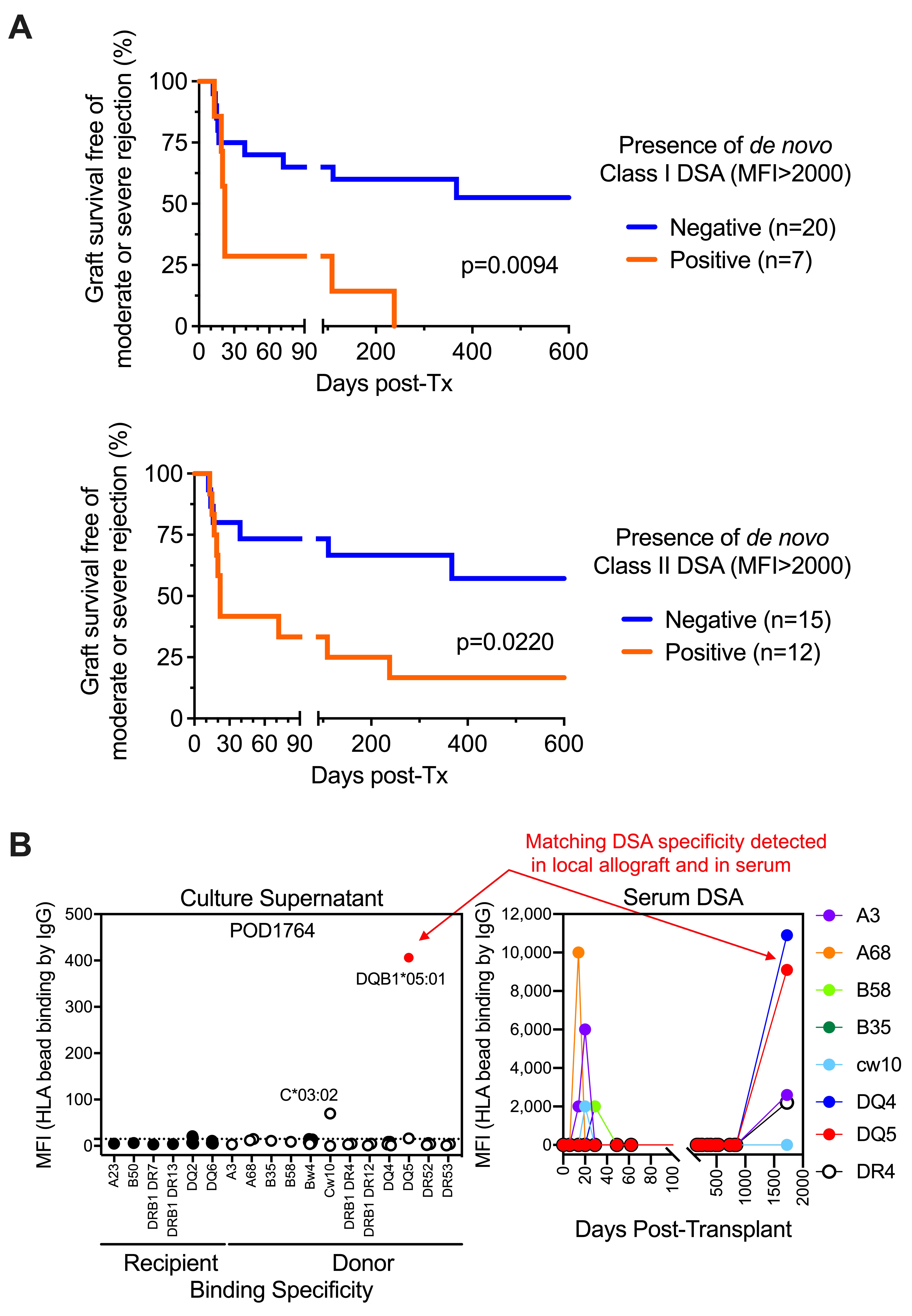


**Supplemental Figure 4.** The fraction of clones per sample with an average v gene mutation frequency >2% by POD is shown in peripheral blood and ileum allograft. The median fraction of mutated clones among adult deceased donors is shown by the dashed lines. Individuals are marked as in Figure 4. Green markers indicate pre-Tx samples (samples taken at POD0).


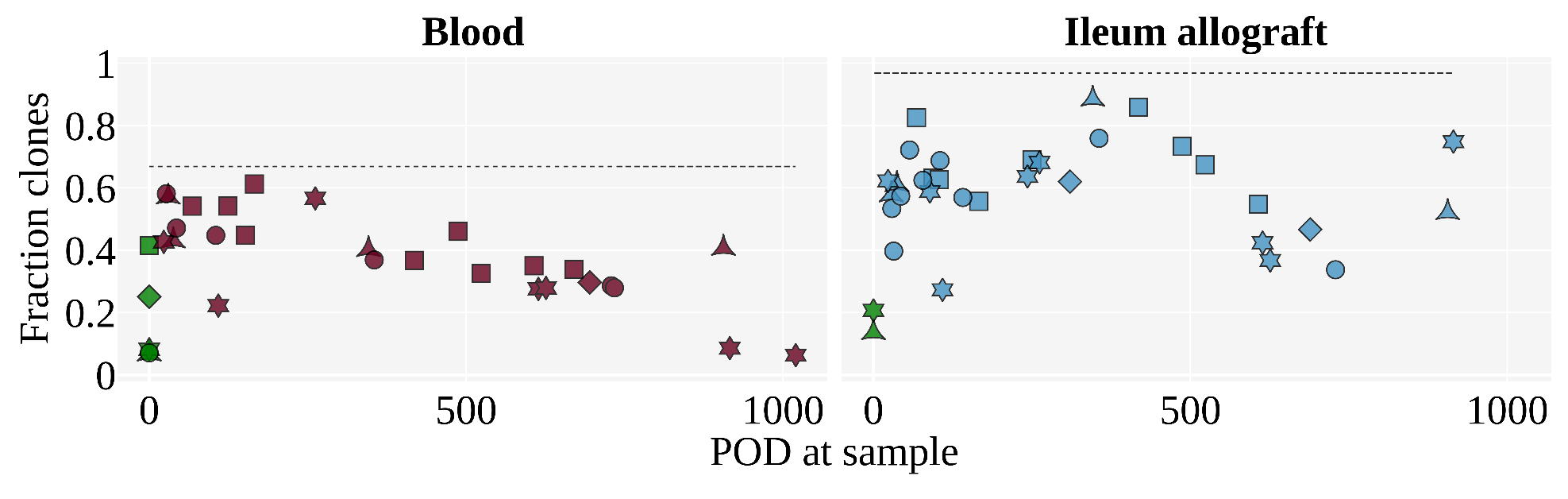


**Supplemental Figure 5.** Clumpiness is a metric quantifying the extent to which specific metadata labels are clumped together in branches of a lineage. Clumpiness values range from 0 to 1. A clumpiness value of 1 between two labels means they are perfectly mixed within a lineage (always found together on all of the branches of a lineage), while a clumpiness value of 0 means the two labels are always on separate branches. Likewise, a clumpiness value of 0.5 means the labels are somewhat mixed within the lineage (the two labels are found together on some of the same branches, but are also found on other branches without the other label).

**
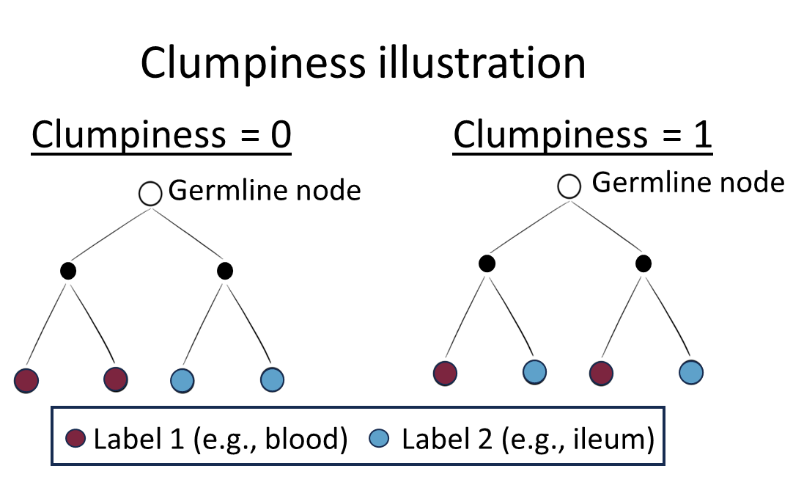
**

**Supplemental Figure 6.** Pediatric transplant patients exhibit increased trafficking between the blood and ileum allograft among mutated (A) and trunk (B) clones compared to adult deceased donor controls. The median clumpiness per individual for a given pair of tissues is shown. In panel (A), clones were filtered for having 3 or more sequenced nodes in their lineages, being mutated (average gene mutation frequency >2%), and being sampled in both tissues that were compared. In panel (B), clones were filtered for having 3 or more sequenced nodes, having a trunk (defined as having at least 5 shared mutations in 85% of a lineage’s sequenced nodes; see Methods), and being sampled in both tissues that were compared. Only medians with greater than 5 clones were included. The Mann-Whitney U test was performed to determine statistical significance (*p<0.05). Individuals are marked as in Figure 4.


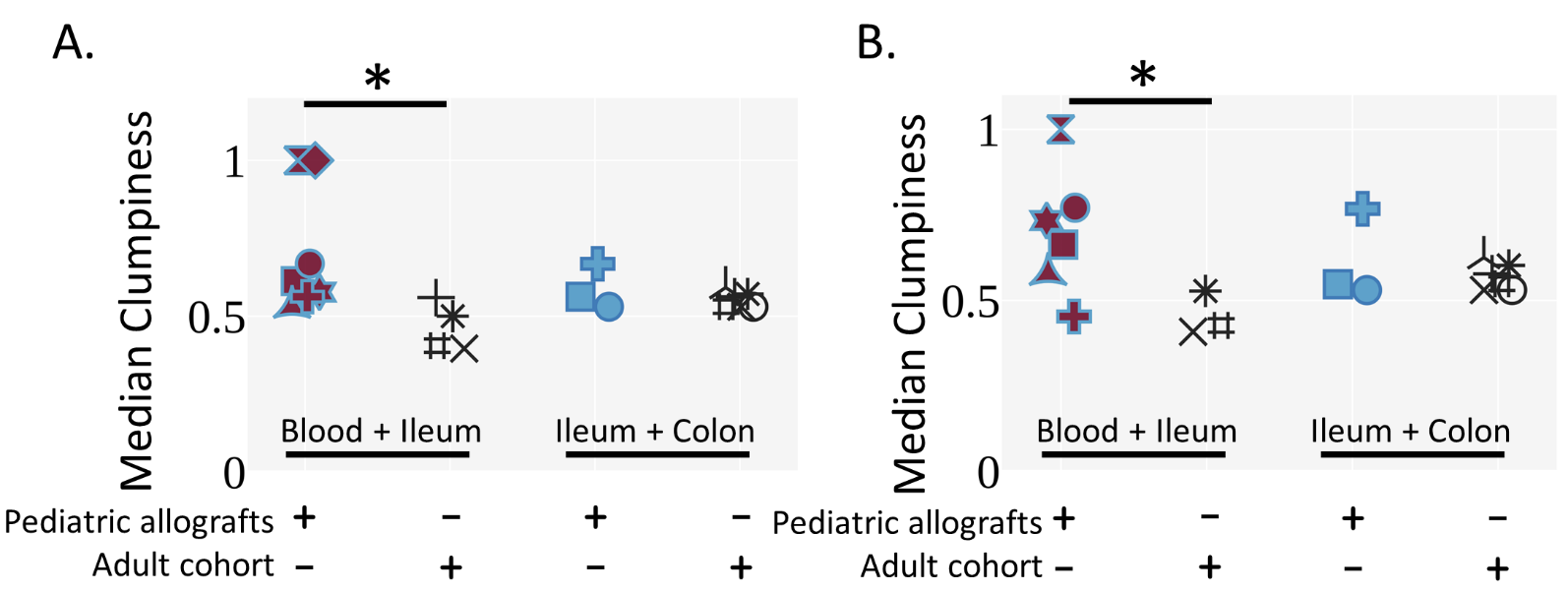


**Supplemental Figure 7.** Median clumpiness between the blood and ileum allograft (or pre-Tx ileum for POD0) per individual by POD was shown. Clones were filtered for having 3 or more unique sequences, and being sampled in both tissues that were compared. Only medians with greater than 5 clones were included. Median clumpiness between the blood and ileum tissues in adult deceased donors is shown by the dotted dashed line. Individuals are marked as in Figure 4

**
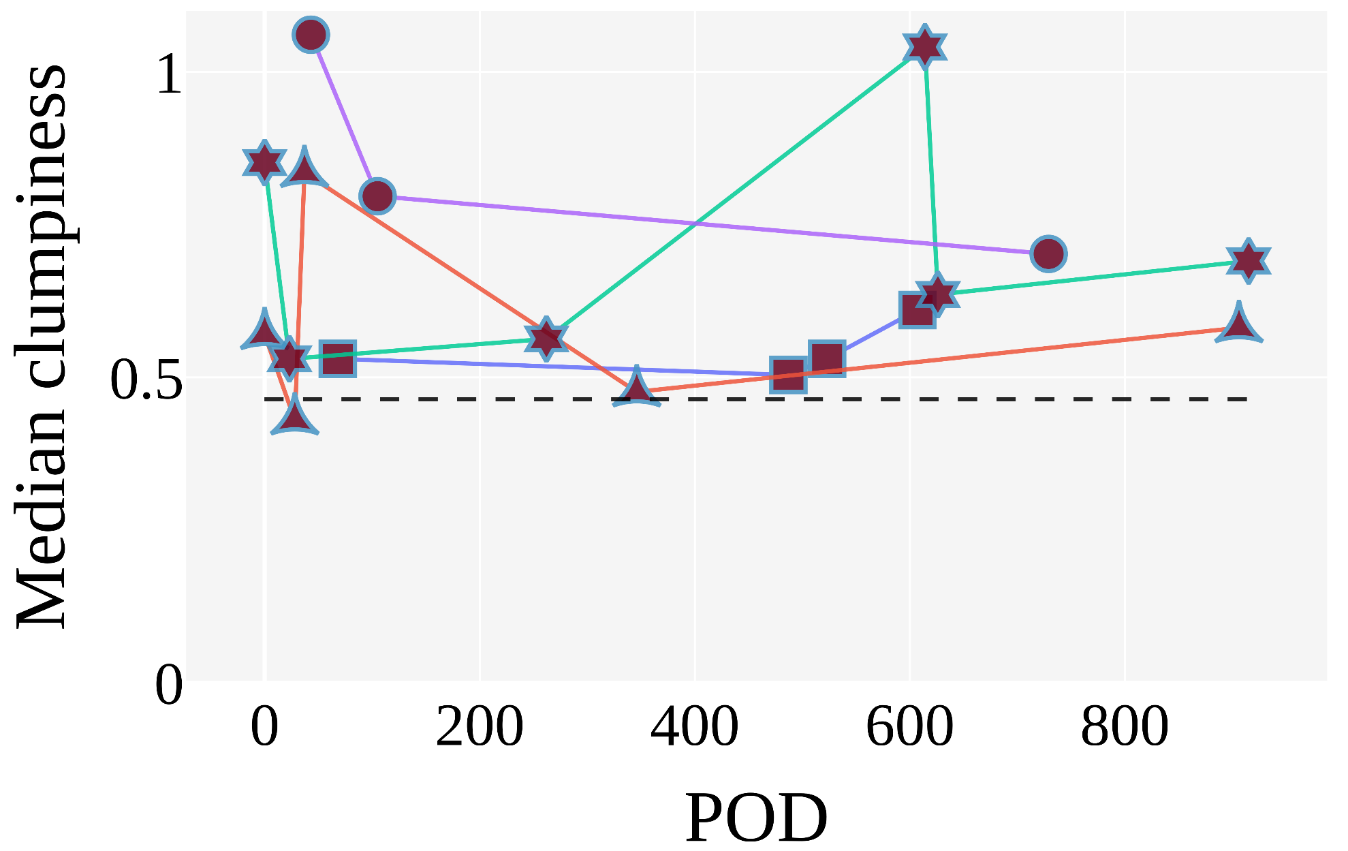
**
